# Dual Combined Real-Time Reverse Transcription Polymerase Chain Reaction Assay for the Diagnosis of Lyssavirus Infection

**DOI:** 10.1371/journal.pntd.0004812

**Published:** 2016-07-05

**Authors:** Laurent Dacheux, Florence Larrous, Rachel Lavenir, Anthony Lepelletier, Abdellah Faouzi, Cécile Troupin, Jalal Nourlil, Philippe Buchy, Herve Bourhy

**Affiliations:** 1 Institut Pasteur, Lyssavirus Dynamics and Host Adaptation Unit, National Reference Centre for Rabies, WHO Collaborating Center for Reference and Research on Rabies, Paris, France; 2 Institut Pasteur du Maroc, Medical Virology and BSL3 Laboratory, Casablanca, Morocco; 3 Institut Pasteur du Cambodge, Virology Unit, Phnom Penh, Cambodia; Wistar Institute, UNITED STATES

## Abstract

The definitive diagnosis of lyssavirus infection (including rabies) in animals and humans is based on laboratory confirmation. The reference techniques for *post-mortem* rabies diagnosis are still based on direct immunofluorescence and virus isolation, but molecular techniques, such as polymerase chain reaction (PCR) based methods, are increasingly being used and now constitute the principal tools for diagnosing rabies in humans and for epidemiological analyses. However, it remains a key challenge to obtain relevant specificity and sensitivity with these techniques while ensuring that the genetic diversity of lyssaviruses does not compromise detection. We developed a dual combined real-time reverse transcription polymerase chain reaction (combo RT-qPCR) method for pan-lyssavirus detection. This method is based on two complementary technologies: a probe-based (TaqMan) RT-qPCR for detecting the *RABV* species (pan-RABV RT-qPCR) and a second reaction using an intercalating dye (SYBR Green) to detect other lyssavirus species (pan-lyssa RT-qPCR). The performance parameters of this combined assay were evaluated with a large panel of primary animal samples covering almost all the genetic variability encountered at the viral species level, and they extended to almost all lyssavirus species characterized to date. This method was also evaluated for the diagnosis of human rabies on 211 biological samples (positive n = 76 and negative n = 135) including saliva, skin and brain biopsies. It detected all 41 human cases of rabies tested and confirmed the sensitivity and the interest of skin biopsy (91.5%) and saliva (54%) samples for *intra-vitam* diagnosis of human rabies. Finally, this method was successfully implemented in two rabies reference laboratories in enzootic countries (Cambodia and Morocco). This combined RT-qPCR method constitutes a relevant, useful, validated tool for the diagnosis of rabies in both humans and animals, and represents a promising tool for lyssavirus surveillance.

## Author Summary

Rabies is an infectious disease of humans and other mammals caused by lyssaviruses. It has one of the highest mortality rates of any infectious disease. Rabies has been known since Antiquity, but it continues to cause approximately 59,000 human deaths per year, particularly in low-income areas in Asia and Africa, where it is still a neglected disease. In these regions, rabies surveillance remains limited, leading to considerable underreporting of rabies cases in humans and animals. One of the principal obstacles to effective surveillance is the difficulty confirming human rabies cases. Clinical diagnosis remains challenging, so all cases must be confirmed by laboratory tests. We describe here the development and validation of a molecular diagnostic tool for lyssavirus infection based on the detection of viral RNA. We evaluated this technique against one of the largest panels of biological samples from animals and humans ever assembled. It was found to be useful and practicable in national reference centers in enzootic regions.

## Introduction

Rabies is an almost invariably fatal form of acute progressive encephalomyelitis that kills an estimated 59,000 humans each year, mostly in low-income Asian and African countries [1]. This zoonosis is transmitted to humans by rabid animals biting, scratching or licking mucous membranes or damaged skin. Moreover, some cases of human-to-human transmission have been described following the transplantation of organs or tissues from donors with undiagnosed rabies [2, 3], and exceptional laboratory cases of human rabies following aerosol contamination [4, 5].

The principal etiological agent, responsible for almost all human rabies cases, is *Rabies virus* (RABV), the prototype species of the genus *Lyssavirus* in the family *Rhabdoviridae* [6, 7]. Thirteen other species of this genus have been identified: *Lagos bat virus* (LBV), *Mokola virus* (MOKV), *Duvenhage virus* (DUVV), *European bat lyssavirus 1* (EBLV-1) and *2* (EBLV-2), *Australian bat lyssavirus* (ABLV), *West Caucasian bat virus* (WCBV), *Irkut virus* (IRKV), *Aravan virus* (ARAV), *Khujand virus* (KHUV), *Shimoni bat virus* (SHIBV), *Ikoma lyssavirus* (IKOV) and *Bokeloh bat lyssavirus* (BBLV) [8]. Another new potential lyssavirus species, Lleida bat lyssavirus, (LLBV) has also recently been identified in bats in Spain [9]. Most of these viruses were isolated from bats, suggesting that lyssaviruses may have originated in Chiroptera [10].

RABV is the lyssavirus with the widest distribution worldwide, and with the broadest spectrum of vectors or animal reservoir hosts from the orders Carnivora and Chiroptera. The domestic dog is a major reservoir host of RABV, implicated in almost all cases of human rabies [7, 11]. However, several other animal species can act as reservoirs for RABV transmission, depending on the geographic area considered. RABV displays also broad genetic diversity, mainly depending of animal hosts and geographic origin.

In animals, rabies is diagnosed *post-mortem*, on a brain specimen, with the fluorescent antibody test (FAT), the designated reference technique of the World Health Organization (WHO) and the Organization for Animal Health (OIE) [7, 12]. This method is also the gold standard for the *post-mortem* diagnosis of human rabies. Many molecular methods have been developed for the *intra-vitam* diagnosis of human rabies. These methods include reverse transcription (RT)-PCR [13–16] and real-time RT-PCR (RT-qPCR) [14, 17–20], but none of the techniques developed to date can deal with the full diversity of the *Lyssavirus* genus and/or have been validated for use with all animal and human specimens collected in the field.

We describe here the development of a one-step pan-lyssavirus detection technique, based on a dual combined real-time reverse-transcription PCR assay (combo RT-qPCR). This assay includes a pan-RABV RT-qPCR probe-based technique able to detect all representatives of the broad genetic diversity of RABV, using two degenerate TaqMan probes. A SYBR Green RT-qPCR assay carried out in parallel can detect all the other lyssaviruses tested, in addition to some RABV isolates. This combo RT-qPCR dedicated to pan-lyssavirus detection assay was found to have high sensitivity and specificity and was able to detect a large panel of animal lyssavirus samples. It also provided accurate *post-mortem* or *intra-vitam* diagnoses of human rabies. The pan-RABV RT-qPCR was then evaluated in two national reference laboratories in enzootic countries, where its utility and efficacy for rabies diagnosis and surveillance were demonstrated.

## Materials and Methods

### Ethics statement

Human samples were obtained from routine diagnostic activities at the National Reference Center for Rabies (NCR-R) of Institut Pasteur, Paris, at the Institut Pasteur du Cambodge (Phnom Penh, Cambodia), at the Institut Pasteur du Maroc (Casablanca, Morocco) or from the NCR-R repository. The NCR-R repository has been registered for research purposes and declared, in accordance with French regulations (article L.1243-3 in relation to the French Public Health Code), to both the French Ministry for Research and a French Ethics Committee, both of which approved and registered the biobank (declaration number DC-2009-1067; collection No. 12).

### Primer design

A region of the polymerase (L) gene (nucleotide positions 7170 to 7419 according to the Pasteur virus genome; GenBank accession number M13215) conserved among lyssavirus genomes was selected as the target of the combo RT-qPCR technique, based on the results of previous studies [15, 21]. This target sequence was amplified from a selected panel of lyssavirus isolates by a previously described RT-hnPCR method [15], and the PCR products were Sanger sequenced and analyzed with Sequencher 5.0 (Gene Codes Corporation) software. The probes and primers of the combo RT-qPCR assay were designed on the basis of a ClustalX 2.1 multiple nucleotide alignment [22]. All primers were designed with OligoAnalyzer 3.1 software, available from http://eu.idtdna.com/calc/analyzer, and checked *in silico* for the formation of hairpins, self-dimers and hetero-dimers and sequency analogy, by blast analysis (BlastN on the NCBI database).

### Viruses and biological samples from animals and humans

The lyssavirus isolates analyzed were used as viral suspensions or as naturally (original brain samples from rabid animals) or experimentally (brains of newborn suckling mice inoculated with the original infected samples) infected animal tissues. All were obtained from the NRC-R or from the WHO Collaborating Center for Reference and Research on Rabies archive, both located at Institut Pasteur, Paris, France, and from the Institut Pasteur du Cambodge at Phnom Penh, Cambodia. Viral suspensions were generated with baby hamster kidney cells (BSR cells) [23], and titrated on the same cells with five-fold serial dilutions, the results being expressed as fluorescent focus units per mL (FFU/mL). Animal brain samples that had tested negative for rabies virus with the FAT were also obtained from the NRC-R.

Human samples were obtained from routine diagnostic activities at the NCR-R of Institut Pasteur, Paris, at the Institut Pasteur du Cambodge (Phnom Penh, Cambodia), at the Institut Pasteur du Maroc (Casablanca, Morocco) or from the NCR-R repository.

### RNA isolation

Total RNA was extracted from titrated viral suspensions with TRI Reagent LS (Molecular Research Center, Cincinnati, Ohio, USA) after the serial dilution of supernatants of infected cell cultures (BSR cells) [23] in a dilution solution prepared from brain specimens from rabies-negative dogs. These specimens were homogenized in culture medium (DMEM) and clarified by centrifugation at 3000 rpm for 5 minutes. For each virus stock previously titrated, serial 10-fold dilutions were prepared in order to obtain from 1x10^6^ to 1 fluorescent focus-forming units (FFU) in a final volume of 0.2 mL for extraction. Extractions were performed in accordance with the kit manufacturer’s protocol. We extracted total RNA from negative and positive (naturally or experimentally infected) animals in TRI Reagent (Molecular Research Center, Cincinnati, Ohio, USA), in accordance with the manufacturer’s instructions.

For human samples and depending of their availability, at least one type of sample (among skin biopsy, saliva and cerebrospinal fluid or CSF) was used per patient for total RNA extraction, as previously described [15]. Briefly, skin biopsy samples were dissociated with sterile scissors and incubated in 180 μL of ALT tissue lysis buffer and 20 μL of proteinase K (Qiagen, Courtaboeuf, France) at 37°C for 3 h, with gentle shaking. The resulting suspension was then mixed with 0.8 mL of TRI Reagent LS (Molecular Research Center, Cincinnati, Ohio, USA) and RNA was extracted according to the manufacturer’s instructions. RNA was extracted from saliva samples, collected using saliva swabs or directly after spiting into a tube (a volume of 0.2 mL was used in this latter case) or from CSF samples (0.2 mL), with TRI Reagent LS (Molecular Research Center, Cincinnati, Ohio, USA), using 2 μL glycogen (5 mg/mL; Life Technologies, Saint Aubin, France) as a coprecipitant. RNA was finally dissolved in 50 μL of nuclease-free water and stored at -70°C until use.

### Preparation of cloned-target plasmids

Viral RNA was extracted from selected RABV isolates (*n* = 9) and from one or two representative LBV, MOKV, DUVV, EBLV-1, EBLV-2 and ABLV lyssaviruses, reverse-transcribed and amplified with the PVO5m/PVO9 primers, as previously described [15]. The PCR products were then inserted into the Topo TA vector according to the manufacturer’s recommendations (Life Technologies, Saint Aubin, France). For the other species and isolates of lyssavirus (WCBV, ARAV, KHUV, IRKV, SHIBV, IKOV, Ozernoe and BBLV), which were not available at the time of this study, the nucleotide region corresponding to the PVO5m/PVO9 fragment was synthesized (Eurofins Genomics, Ebersberg, Germany) based on the reference sequences in GenBank. This region was then inserted into the Topo TA vector, as described above. A large-scale plasmid preparation procedure was carried out and the plasmids obtained were used as a DNA matrix for determining the efficiency and the limit of quantification of the combo RT-qPCR assay.

### Pan-RABV RT-qPCR assay

This one-step, probe-based real-time RT-PCR assay (pan-RABV RT-qPCR) was performed with the Superscript III Platinum One-Step RT-qPCR kit (Life Technologies, Saint Aubin, France), as recommended by the manufacturer, with only minor modifications. Real-time PCR, which was optimized for a final reaction volume of 20 μL, was performed with 10 μL 2x Reaction Mix, 1.5 μL nuclease-free water, 1 μL of each primer Taq3long and Taq17revlong (10 μM), 0.4 μL SuperScript III RT/Platinum *Taq* Mix, 0.3 μL of each probe RABV4 and RABV5 (10 μM), 0.25 μL MgSO_4_ (50 mM), 0.2 μL RNasin recombinant ribonuclease inhibitor (Promega, Charbonnieres, France), 0.05 μL ROX reference dye and 5 μL RNA template (previously diluted 1:10 in nuclease-free water). Amplification was carried out according to the following program: 1 cycle of heating at 45°C for 15 min and 95°C for 3 min, followed by 40 cycles of 95°C for 15 s and 61°C for 1 min, during which fluorescence values were recorded. All reactions were carried out as technical duplicates in Thermo Scientific 96-well plates (Life Technologies, Saint Aubin, France), with an Applied Biosystems 7500 Real-Time PCR System (Life Technologies, Saint Aubin, France). For each RT-qPCR, a quantification cycle number (C_q_) was determined as the PCR cycle number at which the fluorescence of the reaction exceeded a value considered to be significantly higher than background by the software associated with the Applied Biosystems 7500 Real-Time PCR System (Life Technologies, Saint Aubin, France). The efficiency, slope and correlation coefficient (R^2^) were also determined with this software. All reactions were carried out as technical duplicates. A cutoff ≥ 38 was defined for negative results.

### Pan-lyssavirus RT-qPCR assay

This assay was performed with the SuperScript III Platinum SYBR Green One-Step qRT, as recommended by the manufacturer (Life Technologies, Saint Aubin, France), with the same minor modifications as indicated for the pan-RABV assay. In particular, this real-time RT-PCR was optimized for a final volume of 20 μL, using the same mixture composition and the same amount of diluted sample. The primers used were Taq5long and Taq16revlong and the probes were replaced with nuclease-free water. Amplification was performed on a similar thermocycler, as follows: 15 minutes at 45°C, 3 minutes at 95°C, followed by 40 cycles of 15 seconds at 95°C and 1 minute at 55°C, during which fluorescence values were recorded. After the 40 cycles of amplification, a melting analysis was carried out to check the product amplified by determining its specific melting temperature (increase 0.01°C/s, 55–95°C). As previously indicated, the efficiency, slope and correlation coefficient (R^2^) were also determined with the software associated with Applied Biosystems 7500 Real-Time PCR System (Life Technologies, Saint Aubin, France). All reactions were carried out as technical duplicates. For this assay, a positive reaction was not based on the Cq value but exclusively on the melting temperature (Tm) value and the shape of the melting curve, both compared to positive and negative controls.

### Universal internal control system

The quality of RNA extraction was checked with the heterologous internal universal control system based on *in vitro* transcribed eGFP-RNA described by Hoffmann *et al*. (2006) [24]. Working dilutions containing 2x10^5^ copies/μL in nuclease-free water were prepared and stored as aliquots at -70°C. We directly spiked each sample with a total of 10^6^ copies (5 μL) during the extraction step, after adding the TRI Reagent. We then detected eGFP RNA by RT-qPCR, according to a slightly modified version of the method used in the original study [24]. In particular, the conditions of amplification were identical to those used with the pan-RABV RT-qPCR assay. The mixture composition and thermal conditions were identical to those for the pan-RABV RT-qPCR assay, and we used the EGFP1-F and EGFP2-R primers and the EGFP1-FAM probe. A part of the animal and human samples which were retrospectively analysed in this study (i.e. already extracted before the implementation of this internal control) were not spiked with this internal control.

### Other controls used in the combo RT-qPCR assay

For each assay, we performed two negative controls, with nuclease-free water and a calibrated total RNA suspension (0.2 μg/μL) obtained by extraction from a pool of negative dog brain specimens. Two positive controls for the pan-RABV RT-qPCR were also used, consisting of total RNA extracted from suckling newborn mouse brains infected with the CVS strain and diluted 1:10 in the calibrated RNA suspension (0.2 μg/μL). Serial log_10_ dilutions were tested, and two successive serial dilution points were selected in the linear range of the amplification curve determined. Similar positive controls were carried out for the pan-lyssa RT-qPCR assay with the EBLV-1 lyssavirus (isolate 8918FRA).

### RT hemi-nested PCR (RT-hnPCR)

Reverse transcription was performed, as previously described, in a final volume of 30 μL [15]. We then subjected 2 μL of complementary DNA (cDNA) to amplification by hnPCR, with 10 pmol of primers PVO5m/PVO9 in the first round and primers PVO5m/PVO8 in the second round. The amplification reactions contained 2 U AmpliTaq DNA Polymerase (Life Technologies, Saint Aubin, France), 10 nmol of each nucleotide triphosphate, and 62.5 nmol magnesium, as previously indicated [15].

### International interlaboratory trial evaluation

The combo RT-qPCR assay was evaluated by the NRC-R in 2014, in an interlaboratory trial organized by the European Union reference laboratory for rabies, which is located in Nancy, France [25]. The FAT and RT-hnPCR were also evaluated in parallel in this trial. The test panel consisted of nine anonymous samples of freeze-dried homogenized brains, either uninfected or infected with various lyssavirus species. Details of this trial have been provided elsewhere [25].

## Results

### Probe and primer design

**Fig 1:**
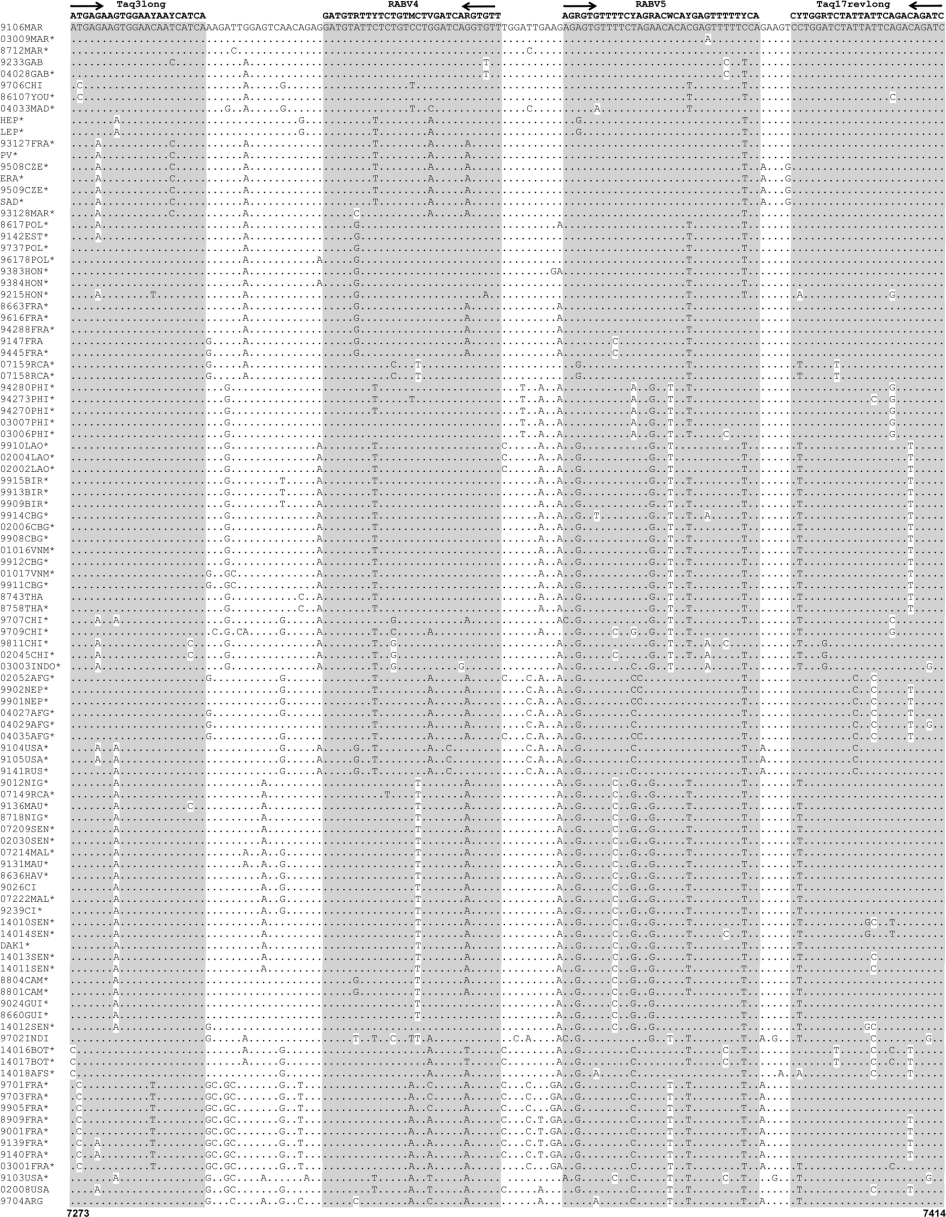
Multiple alignment of the 102 partial polymerase sequences of RABV species, with nucleotide sequences and positions for the Taq3long and Taq17revlong primers and TaqMan hybridization probes RABV4 and RABV5 of the pan-RABV RT-qPCR assay. The oligonucleotide sequence of each primer and probe is indicated in bold, together with its name and an arrow indicating the sense direction. Identity to primer and probe sequences is highlighted in gray. Dots indicate identity to the reference sequence 9106MAR. Positions are indicated according to the reference sequence PV (GenBank accession number M13215). Asterisks indicated partial polymerase sequences obtained in this study. A description of the RABV isolates included in this multiple alignment is provided in S1 Table.

Due to the limited number of nucleotide sequences corresponding to the targeted region of the polymerase gene of lyssavirus available from public databases at the time of this study, a large panel of RABV isolates was selected and sequenced, so as to obtain at least one prototype sequence for each of the principal phyloclades previously defined (S1 Table) [26–28]. We also selected and sequenced a panel of isolates from other lyssavirus species. The probes and primers for the combo RT-qPCR assay were designed from two different sequence datasets. The first dataset contained 102 RABV partial polymerase gene sequences, including 93 newly acquired sequences, and was used to design the pan-RABV RT-qPCR assay, which was based on the Taq3long and Taq17revlong primers and the TaqMan probes RABV4 and RABV5 (Fig 1) (Table 1) (S1 Table). These primers amplified a 143-nucleotide sequence. To complete the evaluation of the spectrum of detection of these primers and probes of the pan-RABV RT-qPCR assay, we analyzed *in silico* another dataset of 91 partial polymerase gene sequences of RABV isolates originated from the New World region (the Americas) and which have been recently published and available in GenBank (S1 Fig) (S2 Table). The genetic diversity observed in this dataset was high, leading to the presence of cumulative mistaches for some isolates.

**Table 1:** Oligonucleotide sequences of primers and probes used in the combo RT-qPCR (combination of pan-RABV and pan-lyssa RT-qPCR assays) and in the internal control eGFP-based RT-qPCR assay.

Application	Reference	Name	Type	Length	Sequence (5’-3’)	Sense	Position
Pan-RABV RT-qPCR assay (TaqMan-based)	This study	Taq3long	Primer	22	ATG AGA AGT GGA AYA AYC ATC A	S	7273–7294^a^
		Taq17revlong	Primer	25	GAT CTG TCT GAA TAA TAG AYC CAR G	AS	7390–7414^a^
		RABV4	Probe (FAM/TAMRA)	29	AAC ACY TGA TCB AGK ACA GAR AAY ACA TC	AS	7314–7342^a^
		RABV5	Probe (FAM/TAMRA)	32	AGR GTG TTT TCY AGR ACW CAY GAG TTT TTY CA	S	7353–7384^a^
Pan-lyssa RT-qPCR assay (SYBR Green-based)	This study	Taq5long	Primer	23	TAT GAG AAA TGG AAC AAY CAY CA	S	7272–7294^a^
		Taq16revlong	Primer	25	GAT TTT TGA AAG AAC TCA TGK GTY C	AS	7366–7390^a^
eGFP internal control assay	Hoffmann et al., 2006 [24]	EGFP1F	Primer	20	GAC CAC TAC CAG CAG AAC AC	S	637–656^b^
		EGFP2R	Primer	19	GAA CTC CAG CAG GAC CAT G	AS	768–750^b^
		EGFP	Probe (VIC/TAMRA)	22	AGC ACC CAG TCC GCC CTG AGC A	S	703–724^b^

^a^ According to the Pasteur virus (PV) RABV genome sequence (GenBank accession number M13215).

^b^ According to the cloning vector pEGFP-1 sequence (GenBank accession number U55761).

**Fig 2:**
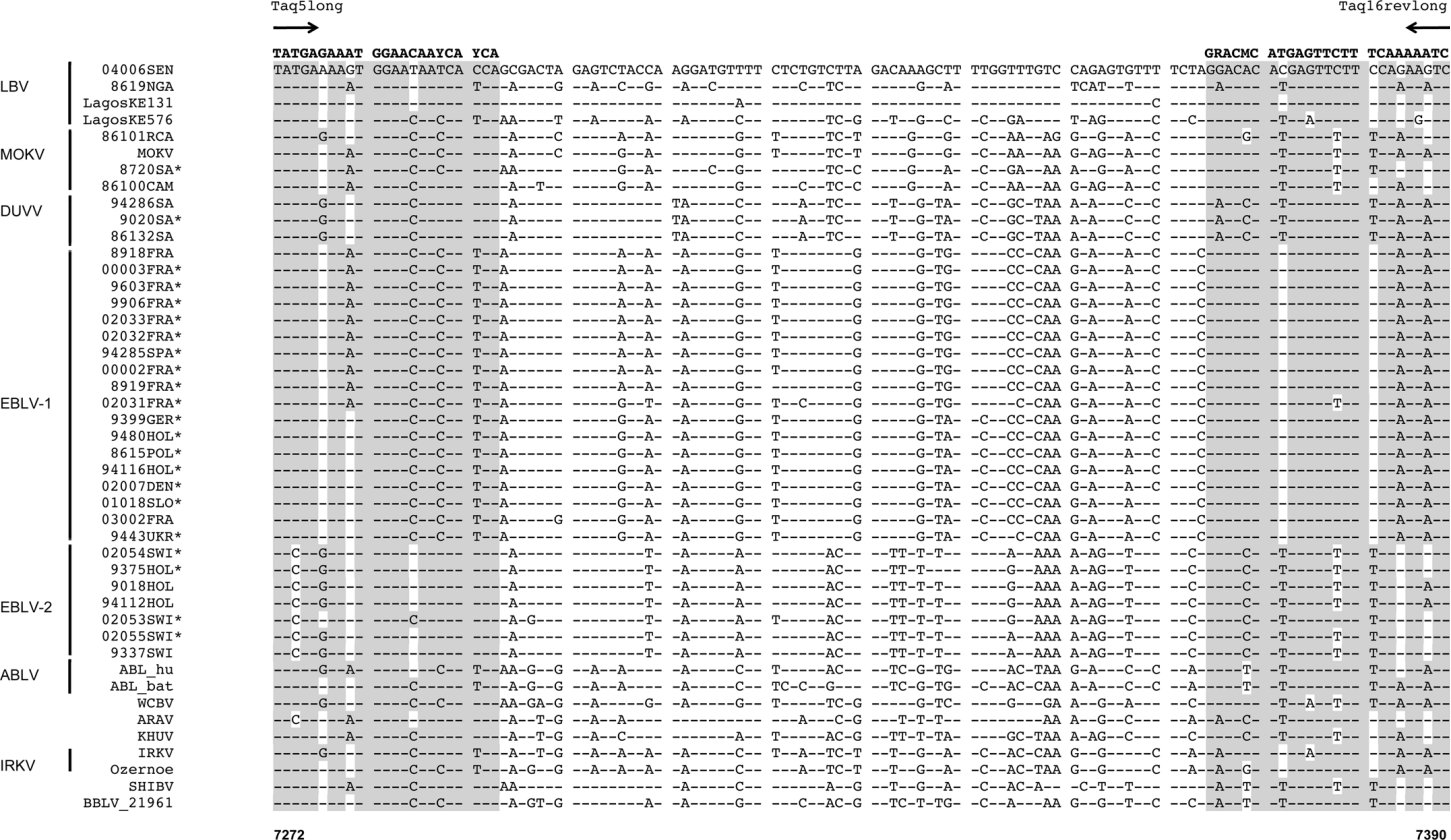
Multiple alignment of 45 partial polymerase sequences from lyssaviruses other than RABV species, with nucleotide sequences and the positions of the Taq5long and Taq16revlong primers (pan-lyssa RT-qPCR). The oligonucleotide sequence of each primer is indicated in bold, together with its name and an arrow indicating the sense direction. Identity to primer sequences is highlighted in gray. Dots indicate identity to the reference sequence 04006SEN. Positions are indicated according to the reference sequence PV (GenBank accession number M13215). Asterisks indicated partial polymerase sequences obtained in this study. Lyssavirus species are indicated on the left side of the figure, and a description of the lyssavirus isolates included in this multiple alignment is provided in S1 Table.

For the pan-lyssa RT-qPCR assay, we used another dataset contained 45 sequences representing all the other lyssavirus species and including 22 new sequences (S1 Table). On the basis of multiple nucleotide alignment data, we defined a single set of primers, Taq5long and Taq16revlong, amplifying a 119-nucleotide region (Fig 2) (Table 1). Due to the high diversity of sequences in all the different lyssavirus species, no consensual probe was designed and we used these primers with an intercalating dye (SYBR Green).

### Determination of the assay acceptance parameters

We first evaluated the intrinsic parameters of the combo RT-qPCR assay with cloned-target plasmids representative of the 14 recognized lyssavirus species (Table 2) [8]. We analyzed nine RABV isolates belonging to different phylogenetic clades and/or lineages with the pan-RABV RT-qPCR assay. The mean efficiency and correlation coefficient (R^2^) values obtained were 97% (±9%) and 0.98 (±0.012), respectively. The limit of quantification ranged from 10 to 10^4^ target copies per assay, with the lowest values obtained for isolates 8693GAB and 03003IND and the highest for isolates 08338GAM and 9105USA. Seventeen other lyssavirus isolates were target-cloned and tested with the pan-lyssa RT-qPCR assay (Table 2). The mean efficiency and R^2^ values obtained were 106% (±25%) and 0.964 (±0.033), respectively. The lowest limit of quantification value was obtained with one isolate of EBLV-1 (02007DAN) and WCBV, at one target copy per assay, and the highest was obtained with one isolate of IRKV, at 10^5^ target copies (efficiency was also lowest for this isolate, at 47%).

**Table 2:** Intrinsic parameters of the combo RT-qPCR assay using target-cloned plasmids.

Species	Isolate	Location	Host	Assay	Slope	R^2^	Efficiency (%)	Limit of quantification	
								Target copy number / reaction	Corresponding Cq max
RABV	CVS^a^	-	Lab strain	Pan-RABV	-3.16	0.980	96	10^2^	37
	PM^b^	-	Lab strain	Pan-RABV	-3.50	0.984	91	10^2^	32
	8693GAB	Gabon	Dog	Pan-RABV	-3.13	0.992	107	10	31
	8743THA	Thailand	Human	Pan-RABV	-3.94	0.991	79	10^3^	35
	9105USA	USA	Arctic fox	Pan-RABV	-3.32	0.945	91	10^4^	34.5
	9147FRA	France	Red fox	Pan-RABV	-3.58	0.995	90	10^2^	35.5
	9704ARG	Argentina	Bat	Pan-RABV	-3.65	0.968	109	10^2^	36.5
	03003IND	Indonesia	Human	Pan-RABV	-3.04	0.976	111	10	34
	08338GAM	Gambia	Dog	Pan-RABV	-3.28	0.995	102	10^4^	35
LBV	8619NGA	Nigeria	Bat	Pan-lyssa	-2.35	0.981	166	10^2^	NA^c^
MOKV	86100CAM	Cameroon	Shrew	Pan-lyssa	-2.36	0.935	165	10^3^	NA
DUVV	86132SA	South Africa	Human	Pan-lyssa	-3.47	0.996	94	10^2^	NA
EBLV-1	8918FRA	France	Bat	Pan-lyssa	-3.42	0.994	96	10^2^	NA
	02007DAN	Denmark	Bat	Pan-lyssa	-3.47	0.998	94	1	NA
EBLV-2	02054SWI	Switzerland	Bat	Pan-lyssa	-3.16	0.993	107	10	NA
	02053SWI	Switzerland	Bat	Pan-lyssa	-3.57	0.999	90	10^2^	NA
	94112HOL	The Netherlands	Bat	Pan-lyssa	-2.99	0.995	116	10^2^	NA
ABLV	9810AUS	Australia	Bat	Pan-lyssa	-3.86	0.998	81	10^2^	NA
WCBV	-	Russia	Bat	Pan-lyssa	-2.15	0.998	192	1	NA
ARAV	-	Kyrgyzstan	Bat	Pan-lyssa	-3.29	0.893	101	10^3^	NA
KHUV	-	Tajikistan	Bat	Pan-lyssa	-3.50	0.944	93	10^2^	NA
IRKV	-	Russia	Bat	Pan-lyssa	-5.95	0.943	47	10^5^	NA
	Ozernoe	Russia	Human	Pan-lyssa	-3.38	0.870	98	10^3^	NA
SHIBV	-	Kenya	Bat	Pan-lyssa	-3.72	0.972	86	10^2^	NA
IKOV	RV2508	Tanzania	Civet	Pan-lyssa	-3.43	0.930	96	10^2^	NA
BBLV	-	Germany	Bat	Pan-lyssa	-3.68	0.945	87	10^2^	NA

^a^ CVS: challenge virus strain

^b^PM: Pitman-Moore strain

^c^ NA: not applicable

We then evaluated the intrinsic parameters of this combo RT-qPCR assay with titrated viral suspensions representative of the main lyssavirus species (Table 3). Four RABV isolates (CVS, 8743THA, 9147FRA and 9704ARG) were tested in the pan-RABV assay. The mean efficiency and R^2^ values were 109% (±23%) and 0.964 (±0.032), respectively, with similar values for the limits of detection and quantification, ranging from 50 to 500 FFU/mL of the initial sample before extraction. At least one representative virus from each of the main other lyssavirus species was analyzed with the pan-lyssa assay (Table 3). The mean efficiency and correlation coefficient values were as good as those for the TaqMan based-assay, at 99% (±8%) and 0.992 (±0.004), respectively. The limit of detection ranged from 38 FFU/mL for one isolate of DUVV (86132SA) to 5,000 FFU/mL for MOKV (isolate 86100CAM) whereas the limit of quantification ranged from 390 FFU/mL for EBLV-1 (isolate 8918FRA) to 15,000 FFU/mL for LBV (isolate 8619NGA).

**Table 3:** Intrinsic parameters of the combo RT-qPCR assay using titrated viral suspensions.

Species	Isolate	Location	Host	Assay	Slope	R^2^	Efficiency	Limit of detection	Corresponding	Limit of quantification
							(%)	(FFU/mL)^a^	Cq max	(FFU/mL)^a^
RABV	CVS^b^	-	Lab strain	Pan-RABV	-2.46	0.900	155	50	36.5	50
	8743THA	Thailand	Human	Pan-RABV	-3.46	0.995	95	50	36	50
	9147FRA	France	Red fox	Pan-RABV	-3.31	0.980	100	50	36	50
	9704ARG	Argentina	Bat	Pan-RABV	-3.64	0.981	88	500	34	500
LBV	8619NGA	Nigeria	Bat	Pan-lyssa	-3.33	0.998	100	1500	NA^c^	15000
MOKV	86100CAM	Cameroon	Shrew	Pan-lyssa	-3.42	0.986	96	5000	NA	5000
DUVV	87020SA	South Africa	Bat	Pan-lyssa	-3.04	0.984	113	500	NA	5000
	86132SA	South Africa	Human	Pan-lyssa	-3.42	0.990	96	38	NA	3850
EBLV-1	8918FRA	France	Bat	Pan-lyssa	-3.67	0.998	87	390	NA	390
EBLV-2	02053SWI	Switzerland	Bat	Pan-lyssa	-3.04	0.994	113	500	NA	500
ABLV	9810AUS	Australia	Bat	Pan-lyssa	-3.59	0.994	90	500	NA	500

^**a**^ Number of fluorescent focus-forming units (FFU) per mL of sample to be extracted.

^b^ CVS: Challenge virus strain

^c^ NA: not applicable

### Determination of the threshold of positivity for pan-RABV RT-qPCR

The cut-off value of the threshold of positivity for the pan-RABV RT-qPCR assay was based on the results obtained from a panel of all negative samples confirmed negative samples (human and animal).

We first analyzed a panel of 40 primary brain samples from 10 different animal species (S3 Table). All had previously tested negative with the reference technique, FAT. All these samples tested in the pan-RABV RT-qPCR assay, provided a mean Cq value of 39.79 associated to a standard deviation (SD) of 0.31, and a minimum Cq value of 38.41.

In parallel, a total of 97 human negative samples (including brain and skin biopsies, saliva and CSF samples) were tested with the pan-RABV RT-qPCR assay (S4 Table). These samples were previously confirmed negative by conventional RT-PCR techniques performed during routine diagnosis by the national reference centre for rabies in France and in Cambodia. The mean Cq value obtained was 39.95 (SD±0.23), with a minimum Cq value of 38.66.

Taking together all these samples (n = 137), we obtained a mean Cq value of 39.92 (SD±0.29). Based on these results we determined the cut-off value for the threshold of detection at 38, corresponding to the mean Cq from which were substracted nearly 6 SD.

### Determination of the sensitivity, specificity and spectrum of detection of the combo RT-qPCR assay for the *post-mortem* diagnosis of animal rabies

Both the pan-RABV and pan-lyssa RT-qPCR assays were evaluated with a large panel of positive animal samples, each previously been confirmed with the reference technique, FAT. We selected 121 RABV isolates considered representative of the main phyloclades, subclades and lineages circulating worldwide (Table 4) (S5 Table) for testing. Eighty corresponded to original brain tissue samples collected from 12 different animal species, whereas the others were amplified by inoculating suckling newborn mice. All the different genetic lineages of RABV tested gave positive results in the pan-RABV RT-qPCR assay, except for three isolates from Senegal belonging to Africa 2 clade (Table 4) (S5 Table). The mean Cq value was 19.52 (SD±5.42), with maximum and minimum Cq values of 36.37 and 9.88, respectively. The sensitivity and spectrum of detection of the pan-lyssa RT-qPCR assay were evaluated with 34 isolates from seven different lyssavirus species: LBV (*n* = 2), MOKV (*n* = 3), DUVV (*n* = 3), EBLV-1 (*n =* 18), EBLV-2 (*n* = 6), ABLV (*n* = 1) and BBLV (*n* = 1) (Table 4) (S5 Table). Five of the samples were primary brain tissues from bats and a cat infected with the EBLV-1 lyssavirus species, and one was from a bat infected with BBLV. Positive detection was obtained for all the isolates tested, with a unique and well-defined dissociation curve, with a melting temperature Tm, centered on 77.75°C (SD±1.06). We also tested 49 RABV isolates with the pan-lyssa RT-qPCR: 41 tested positive, with Cq values below 21. Two of the three samples from Senegal that tested negative in TaqMan assays yielded positive results in this SYBR Green assay. Only two isolates not belonging to the RABV species were detected with the pan-RABV RT-qPCR assay (1 from EBLV-1 and the other from EBLV-2) (Table 4) (S5 Table). Finally, all isolates (*n* = 155) except one (RABV isolate 14011SEN) gave positive results in the combo RT-qPCR compared to the FAT, leading to a high sensitivity (99.3%) (Table 4) (S5 Table).

**Table 4:** Sensitivity and spectrum of detection of the combo RT-qPCR assay using a dataset of positive lyssavirus samples.

	Lyssavirus species	Total Nb	combo RT-qPCR results		
			(Pos no. / Tested no.)		
			pan-RABV	pan-lyssa	combo
			(mean Cq value)		
	RABV	121	118/121 (Cq = 19.52 ±5.24)	41/49	120/121
	LBV	2	0/2	2/2	2/2
	MOKV	3	0/2	3/3	3/3
	DUVV	3	0/2	3/3	3/3
	EBLV-1	18	1/3 (Cq = 30.6)	18/18	18/18
	EBLV-2	6	1/2 (Cq = 31.9)	6/6	6/6
	ABLV	1	ND^a^	1/1	1/1
	BBLV	1	0/1	1/1	1/1
**Total**		155	120/133	75/83	154/155

^a^ ND: Not done

The overall specificity of the combo RT-qPCR assay was complete (100%) when compared to FAT. Indeed, specificity was determined on a panel of 40 primary brain samples from 10 different animal species (S3 Table), which have been previously tested negative with the reference technique, FAT. All these samples tested negative in the pan-RABV RT-qPCR assay, with a Cq value higher than the cut-off value of 38. The same samples were tested with the pan-lyssa RT-qPCR assay and all yielded negative results with non-specific dissociation curves.

### Determination of the sensitivity and specificity of the combo RT-qPCR assay for the diagnosis of human rabies

We assessed the utility of the combo RT-qPCR assay for human rabies diagnosis, using a large collection of positive and negative biological samples collected from 65 different patients (S4 Table). Samples were obtained for *post-mortem* or *intra-vitam* diagnosis in France, Cambodia and Morocco. All samples were tested with the pan-RABV assay and the results obtained were compared with RT-hnPCR, used as the reference technique [15]. Some samples were also tested with the pan-lyssa RT-qPCR assay (S4 Table). In total, 12 samples for *post-mortem* (all brain biopsies) and 199 samples for *intra-vitam* diagnosis were tested with the pan-RABV RT-qPCR assay, including skin biopsy specimens (*n* = 67), saliva (*n* = 120) and CSF (*n* = 12) (Table 5) (S4 Table).

**Table 5:** Evaluation of the intrinsic parameters of the combo RT-qPCR assay for the diagnosis of human rabies, using the RT-hnPCR as the referent method.

	Type of sample	Total number^a^	Combo RT-qPCR result		Mean Cq value ± SD	Sensitivity	Specificity	Positive predicted value	Negative predicted value
			No. pos. samples/Total no. of samples (%)						
			From all patients	From pos. patients					
**Sample**	Skin biopsy	67	43/67 (64.2)	43/47 (91.5)	31.53 ± 4.38	102^b^	100	100	96.3
	Saliva	120	20/120 (16.7)	20/37 (54)	33. 93 ± 3.00	74^c^	100	100	93.4
	CSF	12	3/12 (25)	3/7 (42.9)	25.3 ± 0.14	100	100	100	100
	Brain biopsy	12	10/12 (83.3)	10/10 (100)	21.4 ± 5.56	100	100	100	100
	Total	211	76/211 (36)	76/101 (75.2)	30.70 ± 5.7	92.7	100	100	95.6
**Patient**^d^		65	41/65 (63.1)	41/41 (100)	41/65 (61.5)	100	100	100	100

^a^ Total number includes samples tested in Cambodia, France and Morocco from all patients (negative and positive for rabies).

^b^ One additional skin biopsy sample was detected with the pan-RABV RT-qPCR (n = 43) compared to the RT-hnPCR (n = 42).

^c^ 8 saliva samples from a same patient were not detected and one saliva sample from another patient was detected with the pan-RABV RT-qPCR compared to the RT-hnPCR, leading to a total of n = 20 and n = 27 saliva samples detected for the pan-RABV RT-qPCR and the RT-hnPCR, respectively.

^d^ Including all patients, with rabies-confirmed and negative patients.

For skin specimens, the combo RT-qPCR identified 43 samples as positive, versus 42 for the reference technique, RT-hnPCR, giving a sensitivity of 102%. In total, 41 samples gave positive results in the pan-RABV RT-qPCR, with a mean Cq value of 31.53 (SD±4.38). Two biopsies from patient 28 tested negative in the pan-RABV assay but positive in the pan-lyssa assay (S4 Table). This patient was infected with a rabies virus belonging to the Africa 2 phylogenetic clade [29]. The skin biopsy specimens of patients 2 and 31 tested positive with the pan-RABV RT-qPCR, with Cq values of 35.5 and 33.9, respectively, but negative by RT-hnPCR (S4 Table). For patient 48, a skin biopsy specimen tested negative with the pan-RABV RT-qPCR but gave a weak positive signal with the reference technique.

For saliva, 20 samples tested positive with the pan-RABV RT-qPCR, with a mean Cq value of 33.93 (SD±3.00), versus 27 with the RT-hnPCR technique. All these samples were stored frozen (70°C) before testing. The samples that tested positive by RT-hnPCR included eight saliva samples collected from the same patient (patient 28) from Mali and testing negative with the combo RT-qPCR assay but positive with the reference technique, resulting in a sensitivity of 74% with saliva (Table 5) (S4 Table) [29]. For patient 16, one saliva sample tested positive with the pan-RABV RT-qPCR, with a Cq value of 37.42, but negative with the RT-hnPCR (S4 Table).

For CSF, all results were concordant between the two assays and the mean Cq value was 25.3 (SD±0.14) (Table 5) (S4 Table). In addition, one aspirate of bronchial secretions tested negative with the pan-RABV RT-qPCR but positive by RT-hnPCR (S4 Table) [15].

For *post-mortem* diagnosis, 12 brain specimens were tested and results were positive with both the RT-hnPCR and the pan-RABV RT-qPCR techniques, with a mean Cq value of 21.4 (SD±5.56) (Table 5) (S4 Table).

Compared to the RT-hnPCR, the specificity of the pan-RABV RT-qPCR assay was 100% for all type of negative samples tested, as well as for the pan-lyssa RT-qPCR assay, with non-specific dissociation curves (Table 5) (S4 Table).

The sensitivity and specificity of the combo RT-qPCR were therefore 100% for brain and skin specimens and well as for CSF. A lower sensitivity was obtained for saliva (74%). The specificity was also 100%. No discordance was noticed at the patient level. Overall, 41 of the 65 patients tested gave positive results for both assays.

### Implementation of the pan-RABV RT-qPCR in local settings, in two reference laboratories in enzootic areas

Two national reference laboratories for rabies located in Pasteur Institutes in enzootic areas, Morocco and Cambodia, tested the pan-RABV RT-qPCR assay in their environments. The results for the pan-RABV assay were compared with those for RT-hnPCR, used locally as a reference, for skin biopsies (*n* = 5) and brain biopsies (*n* = 4) in Morocco, and saliva (*n* = 5), skin biopsies (*n* = 35) and brain biopsies (*n* = 4) in Cambodia (S6 Table). The overall sensitivity and specificity were 97.7 and 100%, respectively. Concordance was excellent, with a Kappa value of 0.9 for the two techniques for human diagnosis, with only one saliva sample from Cambodia testing negative in the pan-RABV RT-qPCR assay but weakly positive by RT-hnPCR (S4 and S6 Tables).

### Evaluation of the combo RT-qPCR assay in an international interlaboratory trial

Finally, we evaluated the combo RT-qPCR assay in an interlaboratory trial testing FAT, RT-qPCR and RT-hnPCR on nine anonymous samples; the results obtained were concordant with those expected (S7 Table) [25].

## Discussion

The clinical diagnosis of rabies remains challenging, and is often unreliable, particularly in humans, and confirmation by laboratory methods is therefore required [7, 30]. Laboratory testing is based on the *post-mortem* analysis of brain samples, the gold standard test being the FAT, which can be used together with virus isolation in the rabies tissue culture infectious test (RTCIT) [7, 12, 31, 32]. However, these techniques remain impossible or of limited value for the *intra-vitam* diagnosis of rabies. Alternative new techniques have been proposed over the last two decades, particularly since the advent of molecular techniques, such as reverse transcription-polymerase chain reaction (RT-PCR) [13, 14, 30]. Various protocols have been developed for detecting lyssavirus RNA in different tissue samples by conventional PCR or real-time quantitative PCR (qPCR). Several RT-qPCR techniques for the detection of RABV have been described, based on TaqMan assays targeting a conserved region of nucleoprotein genes [13, 14, 18]. Other assays are dedicated to lyssavirus species, and are designed to discriminate between RABV and EBLV-1 and 2 [14, 17] or to detect RABV, LBV, MOKV and DUVV [19], for example. A SYBR Green-based assay was recently developed for the detection of all lyssavirus species, but it was not tested on field samples or on a broad range of lyssavirus species [33]. Another molecular diagnosis protocol based on a combination of TaqMan-based and SYBR Green-based assays was recently used on animal samples infected with lyssaviruses known to be circulating in Europe [20].

We developed a dual combined RT-qPCR method for pan-lyssavirus detection in samples, for *intra-vitam* diagnosis in humans and *post-mortem* diagnosis in animals. This assay is based on two complementary techniques. The first is probe-based (TaqMan) RT-qPCR for detection of the RABV species (pan-RABV RT-qPCR), whereas the second uses an intercalating dye (SYBR Green) for the detection of other lyssavirus species (pan-lyssa RT-qPCR). The primers of both systems and the probes for the pan-RABV assay were based on a region of the polymerase gene known to be conserved; they were degenerated, to facilitate annealing with a broad spectrum of lyssavirus isolates (Table 1, Figs 1 and 2) [15, 21].

We evaluated the intrinsic parameters of our combo RT-qPCR assay with cloned-target plasmids and titrated viral suspensions. We were able to detect all the lyssavirus species tested with viral suspensions (n = 11) or plasmids (n = 26). The only lyssavirus isolate not tested with this assay was Lleida bat lyssavirus (LLEBV) recently identified in a bat in Spain, for which the sequence of the full genome, including the polymerase gene, was not available [9]. For both assays, the limits of detection were compatible with rabies diagnosis, with values ranged from 38 to 5.10^3^ FFU/mL and from 1 to 10^5^ target copies/mL, for viral suspensions and cloned-target plasmids, respectively.

The performance parameters for this combined assay were evaluated with a large panel of field animal samples representative of the overall genetic variability encountered in RABV species and extending to all lyssavirus species charactarized to date. We tested 121 animal samples with our combo RT-qPCR assay. Only three of the isolates tested (RABV from the Africa 2 clade, originating from Senegal) tested negative in pan-RABV RT-qPCR assay. A combination of at least one mismatch with the forward primer and in each of the two probes was observed for these samples, and more generally for RABV isolates belonging to the Africa 2 clade, potentially accounting for the lower sensitivity for this phylogenetic clade (Fig 1). We tested 50 RABV isolates in our pan-lyssa RT-qPCR system, and 40 gave positive results. This system also detected highly infected RABV samples (Cq<21) and a large proportion of RABV isolates from the Africa 2 clade. Indeed, two of the three isolates from the Africa 2 clade giving negative pan-RABV RT-qPCR results were detected with this assay. The third isolate almost certainly gave negative results due to the small amount of material extracted, given the weak reaction obtained in the RT-hnPCR. All the lyssavirus species tested (34 isolates belonging to 7 different species) with the pan-lyssa RT-qPCR assay were successfully detected, even those associated with a high limit of detection and/or quantification such as LBV and MOKV lyssaviruses. This combo RT-qPCR provides a test with a large spectrum of detection which is not covered by all fluorescent conjugated antibodies commercially available. All the assays gave negative results for all of the negative control materials, indicating a high specificity.

To complete the evaluation of the performance of our pan-RABV RT-qPCR assay, we analyzed *in silico* the targeted nucleotide region of a dataset of RABV isolates originated from New World area, circulating mainly in bats, skunks or raccoons, and which were recently available in GenBank. Indeed, the primers and probes were initially designed based on a limited number of such isolates. A high genetic diversity was found in isolates originated from the Americas, as observed *in silico* with the presence of cumulative mistmaches in the primers and/or probes hybridization regions, which could interfere with their detection (S1 Fig). Although we demonstrated in our study that the pan-RABV RT-qPCR assay allowed the positive detection of different prototype isolates from this region (such as 9704ARG and 9105USA), further evaluation of this assay has to be performed on a larger and more representative dataset of isolates from New World area.

We evaluated the utility of this method for the diagnosis of human rabies, using one of the largest collections of positive and negative biological samples ever assembled. Our test detected all 65 cases of human rabies tested. Strong concordance (98.1%) was observed between the results of RT-qPCR assays and the RT-hnPCR considered as the reference technique. In particular, both the overall sensitivity and specificity of the combo RT-qPCR assay were high, with 92.7% and 100%, respectively. Compared to the RT-hnPCR, this combo RT-qPCR assay performed in our conditions presented the advantage to limit cross-contamination (one-step technique), to be time-saving (two to three times faster) for a similare cost.

We confirmed the interest of skin biopsy and saliva samples for the *intra-vitam* diagnosis of rabies in humans [15, 16]. They allow the diagnosis in 100% of the rabid patients, with 91.5% of skin biopsies and 54% of saliva samples scored positive, in accordance with previous results [15].

This technique requires only small amounts of RNA, with only 3 μL required for the assay. This could be a key advantage in cases in which material is precious or in short supply, such as CSF collected from children or saliva swabs from bats.

We demonstrated the applicability of this test in enzootic areas, in two reference laboratories located in Morocco and Cambodia. Finally this technique was included in an international interlaboratory trial in 2014, in which it provided 100% concordant results [25]. This demonstrates that this test, also not directly applicable in rural settings as most of other molecular methods, can be easily implemented in enzootic countries in laboratories equipped with real-time PCR thermocyclers. In these labs, it could then be considered as a valuable alternative in labs not able to perform FAT or as an interesting confirmatory/back up technique following FAT when needed. This technique could also been a useful complement of rapid and in field diagnostic methods recenlty developed such as rapid immunodiagnostic test (RIDT) (Léchenne et al., in revision in PlosNTD). Compared to other methods of diagnosis for rabies infection, our combo RT-qPCR assay presented the common advantages and limitations of other RT-qPCR methods used for the diagnosis of rabies, and which have been reviewed elsewhere [13, 14, 30, 34].

This study further supports the WHO recommendations asking for the collection of one skin biospsy and/or 3 saliva samples in patients suspected of rabies [7, 30].

## Supporting Information

S1 Checklist STARD Checklist completed. (DOCX)

S1 Table Description of lyssavirus isolates used for the design of primers and probes of the combo RT-qPCR assay (combination of pan-RABV RT-qPCR and pan-lyssa RT-qPCR assays). (DOCX)

S2 Table Description of RABV isolates originated from New World region and used for the multiple alignment in S1 Fig, corresponding to the *in silico* evaluation of primers and probes of the pan-RABV RT-qPCR assay. (XLSX)

S3 Table Results of the analytical specificity of the combo RT-qPCR assay for the *post-mortem* diagnosis of animal rabies. (DOCX)

S4 Table Diagnostic sensitivity and specificity of the combo RT-qPCR compared to the RT-hnPCR for the diagnosis of human rabies. (DOCX)

S5 Table Description of samples used for the *post-mortem* diagnosis of animal rabies and results of the analytical sensitivity of the combo RT-qPCR assay. (DOCX)

S6 Table Results of the implementation of the pan-RABV RT-qPCR in local settings of two national reference laboratories for rabies localized in Morocco and in Cambodia. (DOCX)

S7 Table Evaluation of the combo RT-qPCR assay in an international interlaboratory trial. (DOCX)

S1 Fig Multiple alignment of the dataset of 91 partial polymerase sequences of New World RABV isolates, with nucleotide sequences and positions for primers Taq3long and Taq17revlong and TaqMan hybridization probes RABV4 and RABV5 (pan-RABV RT-qPCR). (PDF)

S1 Flowchart Prototypical STARD diagram completed. (JPG)
