# Supplementary material for: Dual Combined Real-Time Reverse Transcription Polymerase Chain Reaction Assay for the Diagnosis of Lyssavirus Infection
Source: PLoS Negl Trop Dis. 2016 Jul 5;10(7):e0004812. doi: 10.1371/journal.pntd.0004812 (PMC4933377; doi:10.1371/journal.pntd.0004812)
Supplement: S1 Table — (DOCX) [file pntd.0004812.s002.docx]

**S1 Table: Description of lyssavirus isolates used for the design of primers and probes of the combo RT-qPCR assay (combination of pan-RABV RT-qPCR and pan-lyssa RT-qPCR assays).**

| **Species** | **Isolate** | **Host** | **Origin** | **Year of isolation** | **Clade^a^** | **GenBank accession number** |
| --- | --- | --- | --- | --- | --- | --- |
| RABV | 8617POL | Human | Poland | ?^b^ | Cosmopolitan | KU055537 |
| RABV | 8636HAV | Dog | Burkina Faso | 1986 | Africa 2 | KU055538 |
| RABV | 8660GUI | Dog | Guinea | 1986 | Africa 2 | KU055539 |
| RABV | 8663FRA | Dormouse | France | 1986 | Cosmopolitan | KU055540 |
| RABV | 86107YOU | Red fox | Socialist Federal Republic of Yugoslavia (formely) | 1976 | Cosmopolitan | KU055535 |
| RABV | 8712MAR | Human | Morocco | 1986 | Cosmopolitan | KU055541 |
| RABV | 8718NIG | Dog | Niger | 1987 | Cosmopolitan | KU055542 |
| RABV | 8743THA | Human | Thailand | 1983 | Asian | EU293121 |
| RABV | 8758THA | Human | Thailand | 1986 | Asian | KU055545 |
| RABV | 8801CAM | Dog | Cameroon | 1987 | Africa 2 | KU055546 |
| RABV | 8804CAM | Cat | Cameroon | 1988 | Africa 2 | KU055547 |
| RABV | 8909FRA | Bovine | French Guiana | 1989 | Bat | KU055548 |
| RABV | 9001FRA | Dog | French Guiana | 1990 | Bat | EU293113 |
| RABV | 9012NIG | Dog | Niger | 1992 | Africa 2 | KU055550 |
| RABV | 9024GUI | Dog | Guinea | 1990 | Africa 2 | KU055552 |
| RABV | 9026CI | Dog | Ivory Coast | 1990 | Africa 2 | GU815997 |
| RABV | 9103USA | Bat (?) | USA | 1991 | Bat | KU055553 |
| RABV | 9104USA | Skunk | USA | 1991 | Arctic-related | KU055554 |
| RABV | 9105USA | Red fox | USA | 1990 | Arctic-related | GU815998 |
| RABV | 9106MAR | Human | Morocco | 1990 | Cosmopolitan | AY854666 |
| RABV | 9131MAU | Dog | Mauritania | 1991 | Africa 2 | KU055555 |
| RABV | 9136MAU | Goat | Mauritania | 1991 | Africa 2 | KU055556 |
| RABV | 9139FRA | Bovine | French Guiana | 1991 | Bat | KU055557 |
| RABV | 9140FRA | Bovine | French Guiana | 1991 | Bat | KU055558 |
| RABV | 9141RUS | Arctic fox | Russia | 1988-90 | Arctic-related | KU055559 |
| RABV | 9142EST | Raccoon dog | Estonia | 1991 | Cosmopolitan | KU055560 |
| RABV | 9147FRA | Red fox | France | 1991 | Cosmopolitan | EU293115 |
| RABV | 9215HON | Human | Hungary | 1991 | Cosmopolitan | KU055561 |
| RABV | 9233GAB | Dog | Gabon | 1992 | Cosmopolitan | GU815999 |
| RABV | 9239IC | Dog | Ivory Coast | 1992 | Africa 2 | KU055562 |
| RABV | 9383HON | Red fox | Hungary | 1993 | Cosmopolitan | KU055565 |
| RABV | 9384HON | Red fox | Hungary | 1993 | Cosmopolitan | KU055566 |
| RABV | 93127FRA | Vaccinal strain (Pasteur virus) | France | 1993 | Cosmopolitan | KU055563 |
| RABV | 93128MAR | Vaccinal strain (Pasteur virus) | Morocco | ? | Cosmopolitan | GU815994 |
| RABV | 9445FRA | Red fox | France | 1994 | Cosmopolitan | KU055575 |
| RABV | 94270PHI | Dog | Philippines | 1994 | Asian | KU055569 |
| RABV | 94273PHI | Dog | Philippines | 1994 | Asian | KU055570 |
| RABV | 94280PHI | Dog | Philippines | 1994 | Asian | KU055571 |
| RABV | 94288FRA | Red fox | France | 1994 | Cosmopolitan | KU055573 |
| RABV | 9508CZE | Vaccinal strain (SAD virus) | Czech Republic | 1994 | Cosmopolitan | KU055577 |
| RABV | 9509CZE | Vaccinal strain (SAD virus) | Czech Republic | 1994 | Cosmopolitan | KU055578 |
| RABV | 9616FRA | Sheep | France | 1996 | Cosmopolitan | KU055580 |
| RABV | 96178POL | Red fox | Poland | 1994 | Cosmopolitan | KU055581 |
| RABV | 9701FRA | Bovine | French Guiana | 1997 | Bat | KU055582 |
| RABV | 9702IND | Human | India | 1997 | Indian subcontinent | AY854665 |
| RABV | 9703FRA | Bovine | French Guiana | 1997 | Bat | KU055583 |
| RABV | 9704ARG | Bat (*Tadarida brasiliensis*) | Argentina | 1997 | Bat | EU293116 |
| RABV | 9706CHI | Human (vaccinal strain) | China | 1997 | Cosmopolitan | AY854663 |
| RABV | 9707CHI | Human | China | 1980 | Asian | KU055584 |
| RABV | 9709CHI | Dog | China | 1986 | Asian | KU055585 |
| RABV | 9737POL | Raccoon dog | Poland | 1997 | Cosmopolitan | GU816002 |
| RABV | 9811CHI | Dog | China | 1998 | Asian | GU815995 |
| RABV | 9901NEP | Dog | Nepal | 1998 | Arctic-related | KU055586 |
| RABV | 9902NEP | Goat | Nepal | 1998 | Arctic-related | KU055587 |
| RABV | 9905FRA | Bovine | French Guiana | 1999 | Bat | KU055588 |
| RABV | 9908CBG | Dog | Cambodia | 1999 | Asian | KU055590 |
| RABV | 9909BIR | Dog | Myanmar | 1999 | Asian | KU055591 |
| RABV | 9910LAO | Dog | Laos | 1999 | Asian | KU055592 |
| RABV | 9911CBG | Dog | Cambodia | 1998 | Asian | KU055593 |
| RABV | 9912CBG | Dog | Cambodia | 1998 | Asian | KU055594 |
| RABV | 9913BIR | Dog | Myanmar | 1999 | Asian | KU055595 |
| RABV | 9914CBG | Dog | Cambodia | 1997 | Asian | KU055596 |
| RABV | 9915BIR | Dog | Myanmar | 1999 | Asian | KU055597 |
| RABV | 01016VNM | Dog | Vietnam | 2001 | Asian | KU055495 |
| RABV | 01017VNM | Dog | Vietnam | 2001 | Asian | KU055496 |
| RABV | 02002LAO | Dog | Laos | 2002 | Asian | KU055498 |
| RABV | 02004LAO | Dog | Laos | 2002 | Asian | KU055499 |
| RABV | 02006CBG | Dog | Cambodia | 1998 | Asian | KU055500 |
| RABV | 02008USA | Bat (?) | USA | ? | Bat | AY854669 |
| RABV | 02030SEN | Dog | Senegal | 2002 | Africa 2 | KU055502 |
| RABV | 02045CHI | Dog | China | 1989 | Asian | KU055506 |
| RABV | 02052AFG | Dog | Afghanistan | 2002 | Arctic-related | KU055507 |
| RABV | 03001FRA | Dog | French Guiana | 2003 | Bat | KU055511 |
| RABV | 03003INDO | Dog | Indonesia | 2003 | Asian | KU055512 |
| RABV | 03006PHI | Dog | Philippines | 2000 | Asian | KU055513 |
| RABV | 03007PHI | Dog | Philippines | 2001 | Asian | KU055514 |
| RABV | 03009MAR | Dog | Morocco | 2003 | Cosmopolitan | KU055515 |
| RABV | 04027AFG | Dog | Afghanistan | 1996 | Arctic-related | KU055516 |
| RABV | 04028GAB | Cat | Gabon | 2004 | Cosmopolitan | KU055517 |
| RABV | 04029AFG | Dog | Afghanistan | 2004 | Arctic-related | KU055518 |
| RABV | 04033MAD | Human | Madagascar | 2004 | Cosmopolitan | KU055519 |
| RABV | 04035AFG | Dog | Afghanistan | 2004 | Arctic-related | KU055520 |
| RABV | 07149RCA | Dog | Central African Republic | 2004 | Africa 2 | KU055521 |
| RABV | 07158RCA | Dog | Central African Republic | 2006 | Cosmopolitan | KU055522 |
| RABV | 07159RCA | Dog | Central African Republic | 2006 | Cosmopolitan | KU055523 |
| RABV | 07209SEN | Human | Senegal | 2007 | Africa 2 | KU055524 |
| RABV | 07214MAL | Dog | Mali | 2007 | Africa 2 | KU055525 |
| RABV | 07222MAL | Dog | Mali | 2007 | Africa 2 | KU055526 |
| RABV | 14010SEN | Human | Senegal | 2001 | Africa 2 | KU055527 |
| RABV | 14011SEN | Cat | Senegal | 2004 | Africa 2 | KU055528 |
| RABV | 14012SEN | Human | Senegal | 2005 | Africa 2 | KU055529 |
| RABV | 14013SEN | Dog | Senegal | 2011 | Africa 2 | KU055530 |
| RABV | 14014SEN | Dog | Senegal | 2011 | Africa 2 | KU055531 |
| RABV | 14016BOT | Wild cat | Botswana | 2009 | Africa 3 | KU055532 |
| RABV | 14017BOT | Honey badger | Botswana | 2009 | Africa 3 | KU055533 |
| RABV | 14018AFS | Cat | South Africa | 2000 | Africa 3 | KU055534 |
| RABV | DAK1 | ? | Senegal | ? | Africa 2 | KU055598 |
| RABV | ERA | Vaccinal strain | - | ? | Cosmopolitan | KU055599 |
| RABV | HEP | Vaccinal strain | - | ? | Cosmopolitan | KU055600 |
| RABV | LEP | Vaccinal strain | - | ? | Cosmopolitan | KU055601 |
| RABV | PV | Vaccinal strain | - | ? | Cosmopolitan | KU055602 |
| RABV | SAD | Vaccinal strain | - | ? | Cosmopolitan | KU055603 |
| ABLV | ABLbat | Bat (*Pteropus* sp.) | Australia | 1996 | - | NC_003243 |
| ABLV | ABLhu | Human | Australia | 1986 | - | AF418014 |
| ARAV | - | Bat (*Myotis blythi*) | Kyrgyzstan | 1991 | - | EF614259 |
| BBLV | 21961 | Bat (*Myotis nattereri*) | Germany | 2010 | - | JF311903 |
| DUVV | 86132SA | Human | South Africa | 1971 | - | EU293119 |
| DUVV | 9020SA | Bat | South Africa | ? | - | KU055551 |
| DUVV | 94286SA | Bat (*Miniopterus* sp.) | South Africa | 1981 | - | EU293120 |
| EBLV-1 | 8615POL | Bat (*Eptesicus serotinus*) | Poland | 1985 | - | KU055536 |
| EBLV-1 | 8918FRA | Bat (*Eptesicus serotinus*) | France | 1989 | - | EU293112 |
| EBLV-1 | 8919FRA | Bat (*Eptesicus serotinus*) | France | 1989 | - | KU055549 |
| EBLV-1 | 9399GER | Bat (*Eptesicus serotinus*) | Germany | 1982 | - | KU055567 |
| EBLV-1 | 9443UKR | Bat (*Vespertilio murinu*s) | Ukraine | 1987 | - | KU055574 |
| EBLV-1 | 9480HOL | Bat (*Eptesicus serotinus*) | The Netherlands | 1987 | - | KU055576 |
| EBLV-1 | 94116HOL | Bat (*Eptesicus serotinus*) | The Netherlands | 1989 | - | KU055568 |
| EBLV-1 | 94285SPA | Bat (*Eptesicus serotinus*) | Spain | 1994 | - | KU055572 |
| EBLV-1 | 9603FRA | Bat (*Eptesicus serotinus*) | France | 1996 | - | KU055579 |
| EBLV-1 | 9906FRA | Bat (*Eptesicus serotinus*) | France | 1999 | - | KU055589 |
| EBLV-1 | 00002FRA | Bat (*Eptesicus serotinus*) | France | 2000 | - | KU055493 |
| EBLV-1 | 00003FRA | Bat (*Eptesicus serotinus*) | France | 2000 | - | KU055494 |
| EBLV-1 | 01018SLO | Bat (*Eptesicus serotinus*) | Slovakia | 2001 | - | KU055497 |
| EBLV-1 | 02007DEN | Bat (?) | Denmark | 1993 | - | KU055501 |
| EBLV-1 | 02031FRA | Bat (*Eptesicus serotinus*) | France | 2002 | - | KU055503 |
| EBLV-1 | 02032FRA | Bat (*Eptesicus serotinus*) | France | 2002 | - | KU055504 |
| EBLV-1 | 02033FRA | Bat (*Eptesicus serotinus*) | France | 2002 | - | KU055505 |
| EBLV-1 | 03002FRA | Bat (*Eptesicus serotinus*) | France | 2003 | - | EU293109 |
| EBLV-2 | 9018HOL | Bat (*Myotis dasycneme*) | The Netherlands | 1986 | - | EU293114 |
| EBLV-2 | 9337SWI | Bat (*Myotis daubentonii*) | Switzerland | 1993 | - | AY854657 |
| EBLV-2 | 9375HOL | Bat (*Myotis dasycneme*) | The Netherlands | 1993 | - | KU055564 |
| EBLV-2 | 94112HOL | Bat (*Myotis dasycneme*) | The Netherlands | 1989 | - | AY854658 |
| EBLV-2 | 02053SWI | Bat (*Myotis daubentonii*) | Switzerland | 2002 | - | KU055508 |
| EBLV-2 | 02054SWI | Bat (*Myotis daubentonii*) | Switzerland | 1993 | - | KU055509 |
| EBLV-2 | 02055SWI | Bat (*Myotis daubentonii*) | Switzerland | 1992 | - | KU055510 |
| IKOV | RV2508 | Civet | Tanzania | 2009 | - | JX193798 |
| IRKV | - | Bat (*Murina leucogaste*r) | Russia | 2002 | - | EF614260 |
| IRKV | Ozernoe | Human | Russia | 2007 | - | FJ905105 |
| KHUV | - | Bat (*Myotis mystacinus*) | Tajikistan | 2001 | - | EF614261 |
| LBV | 8619NGA | Bat (*Eidolon helvum*) | Nigeria | 1956 | - | EU293110 |
| LBV | 0406SEN | Bat (*Eidolon helvum*) | Senegal | 1985 | - | EU293108 |
| LBV | LagKE131 | Bat (*Eidolon helvum*) | Kenya | 2007 | - | EU259198 |
| MOKV | 86100CAM | Shrew | Cameroon | 1974 | - | EU293117 |
| MOKV | 86101RCA | Rodent | Central African Republic | 1981 | - | EU293118 |
| MOKV | 8720SA | Cat | South Africa | 1987 | - | KU055543 |
| MOKV | MOKV | Cat | Zimbabwe | 1981 | - | NC_006429 |
| SHIBV | Shimoni | Bat (*Hipposideros commersoni*) | Kenya | 2009 | - | GU170201 |
| WCBV | - | Bat (*Miniopterus schreibersii*) | Russia | 2002 | - | EF614258 |

^a^ Phylogenetic clades according to [27].

^b^ ?: Not available
