# Supplementary material for: Dual Combined Real-Time Reverse Transcription Polymerase Chain Reaction Assay for the Diagnosis of Lyssavirus Infection
Source: PLoS Negl Trop Dis. 2016 Jul 5;10(7):e0004812. doi: 10.1371/journal.pntd.0004812 (PMC4933377; doi:10.1371/journal.pntd.0004812)
Supplement: S3 Table — (DOCX) [file pntd.0004812.s004.docx]

**S3 Table: Results of the analytical specificity of the combo RT-qPCR assay for the *post-mortem* diagnosis of animal rabies.**

| **Isolate^a^** | **Sample^b^** | **Host** | **Origin** | **Year of isolation^c^** | **Pan-RABV result**  **(TaqMan)^d^** | **Mean value (Cq)^ef^** | **Pan-lyssa result**  **(SYBR Green)^dg^** |
| --- | --- | --- | --- | --- | --- | --- | --- |
| 03-0706FRA | P | Bat | France | 2003 | Neg | und | Neg |
| 03-0816FRA | P | Bat | France | 2003 | Neg | und | Neg |
| 09-0934FRA | P | Cat | France | 2009 | Neg | 39.9 | Neg |
| 09-0957FRA | P | Cat | France | 2009 | Neg | und | Neg |
| 09-0970FRA | P | Cat | France | 2010 | Neg | und | Neg |
| 10-0525FRA | P | Cat | France | 2010 | Neg | und | Neg |
| 10-0533FRA | P | Cat | France | 2010 | Neg | und | Neg |
| 10-0615FRA | P | Cat | France | 2010 | Neg | 39.44 | Neg |
| 10-0621FRA | P | Cat | France | 2010 | Neg | und | Neg |
| 10-0894FRA | P | Cat | France | 2010 | Neg | und | Neg |
| 09-0907FRA | P | Dog | France | 2009 | Neg | 39.14 | Neg |
| 09-0945FRA | P | Dog | France | 2009 | Neg | und | Neg |
| 09-0960FRA | P | Dog | France | 2009 | Neg | 38.41 | Neg |
| 10-0534FRA | P | Dog | France | 2010 | Neg | und | Neg |
| 10-0560FRA | P | Dog | France | 2010 | Neg | 38.5 | Neg |
| 10-0603FRA | P | Dog | France | 2010 | Neg | und | Neg |
| 10-0814FRA | P | Dog | France | 2010 | Neg | und | Neg |
| 10-0913FRA | P | Dog | France | 2010 | Neg | und | Neg |
| 09-0923FRA | P | Fox | France | 2009 | Neg | und | Neg |
| 09-1352FRA | P | Fox | France | 2009 | Neg | und | Neg |
| 10-0466FRA | P | Fox | France | 2010 | Neg | und | Neg |
| 10-0438FRA | P | Fox | France | 2010 | Neg | 39.61 | Neg |
| 10-0602FRA | P | Fox | France | 2010 | Neg | und | Neg |
| 10-0793FRA | P | Bovine | France | 2010 | Neg | und | Neg |
| 10-0084FRA | P | Bovine | France | 2010 | Neg | und | Neg |
| 10-0055FRA | P | Horse | France | 2010 | Neg | und | Neg |
| 09-1318FRA | P | Horse | France | 2009 | Neg | und | Neg |
| 10-0498FRA | P | Sheep | France | 2010 | Neg | und | Neg |
| 10-0536FRA | P | Deer | France | 2010 | Neg | und | Neg |
| 10-0016FRA | P | Badger | France | 2010 | Neg | 39.19 | Neg |
| 10-0527FRA | P | Mongoose | France | 2010 | Neg | und | Neg |
| Negative 07-12* | P | Fox | France | - | Neg | 39.03 | Neg |
| W0716675** | P | Dog | Cambodia | - | Neg | und | ND |
| W0717651** | P | Dog | Cambodia | - | Neg | und | ND |
| W0717666** | P | Dog | Cambodia | - | Neg | und | ND |
| W0611707** | P | Dog | Cambodia | - | Neg | und | ND |
| W0613648** | P | Dog | Cambodia | - | Neg | und | ND |
| W0613651** | P | Dog | Cambodia | - | Neg | und | ND |
| W0614639** | P | Dog | Cambodia | - | Neg | und | ND |
| W0625663** | P | Dog | Cambodia | - | Neg | und | ND |

^a^ * : Samples tested at the NRC-R, Institut Pasteur, Paris, during its participation in the framework of the international interlaboratory trial organized by the European Union reference laboratory for rabies, ** : Samples tested at the Institut Pasteur du Cambodge, Cambodia. All other samples were tested at the NRC-R, Institut Pasteur, Paris.

^b^ P : Primary brain sample

^c^ - : No information available

^d^ Pos : positive, Neg : negative

^e^ Mean Cq value from duplicates

^f^ Und : Cq value >40

^g^ ND : Not done
