# Supplementary material for: Dual Combined Real-Time Reverse Transcription Polymerase Chain Reaction Assay for the Diagnosis of Lyssavirus Infection
Source: PLoS Negl Trop Dis. 2016 Jul 5;10(7):e0004812. doi: 10.1371/journal.pntd.0004812 (PMC4933377; doi:10.1371/journal.pntd.0004812)
Supplement: S4 Table — (DOCX) [file pntd.0004812.s005.docx]

**S4 Table. Diagnostic sensitivity and specificity of the combo RT-qPCR compared to the RT-hnPCR for the diagnosis of human rabies.**

| **Patient^a^** | | | |  | **Sample** | | | | | | | |  | **Final diagnosis^d^** |
| --- | --- | --- | --- | --- | --- | --- | --- | --- | --- | --- | --- | --- | --- | --- |
|  |  |  |  |  |  |  |  |  |  |  |  |  |  |  |
| **Number of patient**  **(code)** | **Sex^b^** | **Age (year)** | **Origin** |  | **Type** | **Pan-lyssa RT-qPCR** | | | |  | **RT-hnPCR** | |  |  |
|  |  |  |  |  |  | **Pan-rabies (TaqMan)** | | **Pan-lyssa (SYBR Green)**^d^**^e^**  **(Tm in °C)^cf^** | **Summary**  **(Total pos. / total number)** |  | **Result^d^** | **Summary**  **(Total pos. / total number)** |  |  |
|  |  |  |  |  |  | **Cq value^c^** | **Result^d^** |  |  |  |  |  |  |  |
|  |  |  |  |  |  |  |  |  |  |  |  |  |  |  |
| 1 (IPP01) | M | 3 | France (ex Gabon) |  | Saliva | 29.5 | Pos | Neg | 2/2 |  | Pos | 2/2 |  | Pos |
|  |  |  |  |  | Saliva | 27.5 | Pos | Neg |  |  | Pos |  |  |  |
| 2 (IPC01) | M | 22 | Cambodia |  | Skin biopsy | 35.5 | Pos | Neg | 1/1 |  | Neg | 0/1 |  | Pos |
|  |  |  |  |  |  |  |  |  |  |  |  |  |  |  |
| 3 (IPC04) | M | 32 | Cambodia |  | Skin biopsy | 32.9 | Pos | ND | 1/1 |  | Pos | 1/1 |  | Pos |
|  |  |  |  |  |  |  |  |  |  |  |  |  |  |  |
| 4 (IPC05) | F | 47 | Cambodia |  | Skin biopsy | 29.8 | Pos | Neg | 1/1 |  | Pos | 1/1 |  | Pos |
|  |  |  |  |  |  |  |  |  |  |  |  |  |  |  |
| 5 (IPM02) | F | 16 | Madagascar |  | Saliva | > 40 | Neg | ND | 0/2 |  | Neg | 0/2 |  | Neg |
|  |  |  |  |  | Saliva | > 40 | Neg | ND |  |  | Neg |  |  |  |
| 6 (IPM04) | H | 44 | Madagascar |  | Skin biopsy | > 40 | Neg | Neg | 0/14 |  | Neg | 0/14 |  | Neg |
|  |  |  |  |  | Skin biopsy | > 40 | Neg | Neg |  |  | Neg |  |  |  |
|  |  |  |  |  | Saliva | > 40 | Neg | ND |  |  | Neg |  |  |  |
|  |  |  |  |  | Saliva | > 40 | Neg | Neg |  |  | Neg |  |  |  |
|  |  |  |  |  | Saliva | > 40 | Neg | ND |  |  | Neg |  |  |  |
|  |  |  |  |  | Saliva | > 40 | Neg | ND |  |  | Neg |  |  |  |
|  |  |  |  |  | Saliva | > 40 | Neg | ND |  |  | Neg |  |  |  |
|  |  |  |  |  | Saliva | > 40 | Neg | Neg |  |  | Neg |  |  |  |
|  |  |  |  |  | Saliva | > 40 | Neg | ND |  |  | Neg |  |  |  |
|  |  |  |  |  | Saliva | > 40 | Neg | ND |  |  | Neg |  |  |  |
|  |  |  |  |  | Saliva | > 40 | Neg | ND |  |  | Neg |  |  |  |
|  |  |  |  |  | Saliva | > 40 | Neg | ND |  |  | Neg |  |  |  |
|  |  |  |  |  | Saliva | > 40 | Neg | ND |  |  | Neg |  |  |  |
|  |  |  |  |  | Saliva | > 40 | Neg | ND |  |  | Neg |  |  |  |
| 7 (IMP05) | H | 52 | Madagascar |  | Skin biopsy | > 40 | Neg | Neg | 0/13 |  | Neg | 0/13 |  | Neg |
|  |  |  |  |  | Skin biopsy | > 40 | Neg | ND |  |  | Neg |  |  |  |
|  |  |  |  |  | Saliva | > 40 | Neg | ND |  |  | Neg |  |  |  |
|  |  |  |  |  | Saliva | > 40 | Neg | ND |  |  | Neg |  |  |  |
|  |  |  |  |  | Saliva | > 40 | Neg | ND |  |  | Neg |  |  |  |
|  |  |  |  |  | Saliva | > 40 | Neg | Neg |  |  | Neg |  |  |  |
|  |  |  |  |  | Saliva | > 40 | Neg | ND |  |  | Neg |  |  |  |
|  |  |  |  |  | Saliva | > 40 | Neg | ND |  |  | Neg |  |  |  |
|  |  |  |  |  | Saliva | > 40 | Neg | ND |  |  | Neg |  |  |  |
|  |  |  |  |  | Saliva | > 40 | Neg | ND |  |  | Neg |  |  |  |
|  |  |  |  |  | Saliva | > 40 | Neg | ND |  |  | Neg |  |  |  |
|  |  |  |  |  | Saliva | > 40 | Neg | ND |  |  | Neg |  |  |  |
|  |  |  |  |  | Saliva | > 40 | Neg | ND |  |  | Neg |  |  |  |
| 8 (IPM06) | F | 68 | Madagascar |  | Skin biopsy | > 40 | Neg | ND | 0/12 |  | Neg | 0/12 |  | Neg |
|  |  |  |  |  | Saliva | > 40 | Neg | ND |  |  | Neg |  |  |  |
|  |  |  |  |  | Saliva | > 40 | Neg | ND |  |  | Neg |  |  |  |
|  |  |  |  |  | Saliva | > 40 | Neg | ND |  |  | Neg |  |  |  |
|  |  |  |  |  | Saliva | > 40 | Neg | ND |  |  | Neg |  |  |  |
|  |  |  |  |  | Saliva | > 40 | Neg | ND |  |  | Neg |  |  |  |
|  |  |  |  |  | Saliva | > 40 | Neg | ND |  |  | Neg |  |  |  |
|  |  |  |  |  | Saliva | > 40 | Neg | ND |  |  | Neg |  |  |  |
|  |  |  |  |  | Saliva | > 40 | Neg | ND |  |  | Neg |  |  |  |
|  |  |  |  |  | Saliva | > 40 | Neg | ND |  |  | Neg |  |  |  |
|  |  |  |  |  | Saliva | > 40 | Neg | ND |  |  | Neg |  |  |  |
|  |  |  |  |  | Saliva | > 40 | Neg | ND |  |  | Neg |  |  |  |
| 9 (H040006) | M | 17 | France |  | Skin biopsy | > 40 | Neg | Neg | 0/3 |  | Neg | 0/3 |  | Neg |
|  |  |  |  |  | Saliva | > 40 | Neg | Neg |  |  | Neg |  |  |  |
|  |  |  |  |  | LCR | > 40 | Neg | ND |  |  | Neg |  |  |  |
| 10 (H070017) | M | 30 | France |  | Saliva | > 40 | Neg | ND | 0/2 |  | Neg | 0/2 |  | Neg |
|  |  |  |  |  | Skin biopsy | > 40 | Neg | ND |  |  | Neg |  |  |  |
| 11 (H070021) | M | 30 | France (Sri-Lanka) |  | Skin biopsy | > 40 | Neg | Neg | 0/3 |  | Neg | 0/3 |  | Neg |
|  |  |  |  |  | Saliva | > 40 | Neg | ND |  |  | Neg |  |  |  |
|  |  |  |  |  | Saliva | > 40 | Neg | Neg |  |  | Neg |  |  |  |
| 12 (H080001) | M | 70 | France (Cap vert) |  | Saliva | > 40 | Neg | Neg | 0/4 |  | Neg | 0/4 |  | Neg |
|  |  |  |  |  | Saliva | > 40 | Neg | ND |  |  | Neg |  |  |  |
|  |  |  |  |  | Saliva | > 40 | Neg | Neg |  |  | Neg |  |  |  |
|  |  |  |  |  | Saliva | > 40 | Neg | ND |  |  | Neg |  |  |  |
| 13 (H080004) | M | 42 | French Guiana |  | Saliva | 28.4 | Pos | Neg | 3/3 |  | Pos | 3/3 |  | Pos |
|  |  |  |  |  | Saliva | 34.3 | Pos | Neg |  |  | Pos |  |  |  |
|  |  |  |  |  | Saliva | 30.3 | Pos | Neg |  |  | Pos |  |  |  |
| 14 (H090011) | M | 9 | France |  | Saliva | > 40 | Neg | ND | 0/3 |  | Neg | 0/3 |  | Neg |
|  |  |  |  |  | Saliva | > 40 | Neg | ND |  |  | Neg |  |  |  |
|  |  |  |  |  | Saliva | > 40 | Neg | Neg |  |  | Neg |  |  |  |
| 15 (H100003) | M | 48 | France |  | LCR | > 40 | Neg | ND | 1/1 |  | Neg | 1/1 |  | Neg |
|  |  |  |  |  |  |  |  |  |  |  |  |  |  |  |
| 16 (H110007) | F | 41 | Portugal (Guinea) |  | Skin biopsy | > 40 | Neg | ND | 18/30 |  | Neg | 17/30 |  | Pos |
|  |  |  |  |  | Saliva | 35.2 | Pos | ND |  |  | Pos |  |  |  |
|  |  |  |  |  | Saliva | 35.7 | Pos | ND |  |  | Pos |  |  |  |
|  |  |  |  |  | Saliva | 36.4 | Pos | ND |  |  | Pos |  |  |  |
|  |  |  |  |  | LCR | > 40 | Neg | ND |  |  | Neg |  |  |  |
|  |  |  |  |  | LCR | > 40 | Neg | ND |  |  | Neg |  |  |  |
|  |  |  |  |  | LCR | > 40 | Neg | Neg |  |  | Neg |  |  |  |
|  |  |  |  |  | LCR | 32.7 | Pos | Neg |  |  | Pos |  |  |  |
|  |  |  |  |  | Skin biopsy | 33 | Pos | ND |  |  | Pos |  |  |  |
|  |  |  |  |  | Skin biopsy | 34.7 | Pos | Neg |  |  | Pos |  |  |  |
|  |  |  |  |  | Skin biopsy | > 40 | Neg | ND |  |  | Neg |  |  |  |
|  |  |  |  |  | Saliva | 31.8 | Pos | Neg |  |  | Pos |  |  |  |
|  |  |  |  |  | Saliva | 33.7 | Pos | ND |  |  | Pos |  |  |  |
|  |  |  |  |  | Saliva | 34 | Pos | Neg |  |  | Pos |  |  |  |
|  |  |  |  |  | Saliva | 31.9 | Pos | Neg |  |  | Pos |  |  |  |
|  |  |  |  |  | Saliva | 36.6 | Pos | Neg |  |  | Pos |  |  |  |
|  |  |  |  |  | Saliva | 35.5 | Pos | Neg |  |  | Pos |  |  |  |
|  |  |  |  |  | Saliva | > 40 | Neg | Neg |  |  | Neg |  |  |  |
|  |  |  |  |  | Saliva | 35.2 | Pos | Neg |  |  | Pos |  |  |  |
|  |  |  |  |  | Saliva | 35.8 | Pos | Neg |  |  | Pos |  |  |  |
|  |  |  |  |  | Saliva | 36.5 | Pos | Neg |  |  | Pos |  |  |  |
|  |  |  |  |  | Saliva | > 40 | Neg | Neg |  |  | Neg |  |  |  |
|  |  |  |  |  | Saliva | 37.1 | Pos | Neg |  |  | Pos |  |  |  |
|  |  |  |  |  | Saliva | 37.09 | Pos | Neg |  |  | Pos |  |  |  |
|  |  |  |  |  | Saliva | > 40 | Neg | ND |  |  | Neg |  |  |  |
|  |  |  |  |  | Saliva | > 40 | Neg | Neg |  |  | Neg |  |  |  |
|  |  |  |  |  | Saliva | > 40 | Neg | Neg |  |  | Neg |  |  |  |
|  |  |  |  |  | Saliva | 37.42 | Pos | Neg |  |  | Neg |  |  |  |
|  |  |  |  |  | Saliva | > 40 | Neg | Neg |  |  | Neg |  |  |  |
|  |  |  |  |  | Saliva | > 40 | Neg | Neg |  |  | Neg |  |  |  |
| 17 (H110009) | M | 56 | France |  | Skin biopsy | > 40 | Neg | ND | 0/6 |  | Neg | 0/6 |  | Neg |
|  |  |  |  |  | Saliva | > 40 | Neg | ND |  |  | Neg |  |  |  |
|  |  |  |  |  | Saliva | > 40 | Neg | ND |  |  | Neg |  |  |  |
|  |  |  |  |  | Saliva | > 40 | Neg | ND |  |  | Neg |  |  |  |
|  |  |  |  |  | Saliva | > 40 | Neg | ND |  |  | Neg |  |  |  |
|  |  |  |  |  | LCR | > 40 | Neg | ND |  |  | Neg |  |  |  |
| 18 (H120004) | F | 41 | France |  | Skin biopsy | > 40 | Neg | ND | 0/2 |  | Neg | 0/2 |  | Neg |
|  |  |  |  |  | Saliva | > 40 | Neg | ND |  |  | Neg |  |  |  |
| 19 (H120009) | M | 4 | France (North Africa) |  | Brain biopsy | > 40 | Neg | ND | 0/1 |  | Neg | 0/1 |  | Neg |
|  |  |  |  |  |  |  |  |  |  |  |  |  |  |  |
| 20 (H120010) | M | 32 | France |  | Saliva | 39.53 et > 40 | Neg | Neg | 0/3 |  | Neg | 0/3 |  | Neg |
|  |  |  |  |  | Saliva | > 40 | Neg | Neg |  |  | Neg |  |  |  |
|  |  |  |  |  | Saliva | > 40 | Neg | Neg |  |  | Neg |  |  |  |
| 21 (H120011) | M | 6 | Greece |  | Skin biopsy | 38.9 | Neg | Neg | 0/5 |  | Neg | 0/5 |  | Neg |
|  |  |  |  |  | LCR | 39.34 | Neg | Neg |  |  | Neg |  |  |  |
|  |  |  |  |  | Saliva | > 40 | Neg | Neg |  |  | Neg |  |  |  |
|  |  |  |  |  | Saliva | > 40 | Neg | Neg |  |  | Neg |  |  |  |
|  |  |  |  |  | Saliva | > 40 | Neg | Neg |  |  | Neg |  |  |  |
| 22 (H120012) | M | 25 | France |  | LCR | > 40 | Neg | Neg | 0/5 |  | Neg | 0/5 |  | Neg |
|  |  |  |  |  | Skin biopsy | > 40 | Neg | Neg |  |  | Neg |  |  |  |
|  |  |  |  |  | Saliva | > 40 | Neg | Neg |  |  | Neg |  |  |  |
|  |  |  |  |  | Saliva | > 40 | Neg | Neg |  |  | Neg |  |  |  |
|  |  |  |  |  | Saliva | > 40 | Neg | Neg |  |  | Neg |  |  |  |
| 23 (H120013) | M | 50 | France |  | Saliva | > 40 | Neg | ND | 0/3 |  | Neg | 0/3 |  | Neg |
|  |  |  |  |  | Saliva | > 40 | Neg | ND |  |  | Neg |  |  |  |
|  |  |  |  |  | Saliva | > 40 | Neg | ND |  |  | Neg |  |  |  |
| 24 (H120014) | M | 40 | France |  | Skin biopsy | > 40 | Neg | Neg | 0/5 |  | Neg | 0/5 |  | Neg |
|  |  |  |  |  | Skin biopsy | > 40 | Neg | Neg |  |  | Neg |  |  |  |
|  |  |  |  |  | Saliva | 38.7 | Neg | Neg |  |  | Neg |  |  |  |
|  |  |  |  |  | Saliva | > 40 | Neg | Neg |  |  | Neg |  |  |  |
|  |  |  |  |  | Saliva | > 40 | Neg | Neg |  |  | Neg |  |  |  |
| 25 (H120017) | M | 67 | France |  | Skin biopsy | > 40 | Neg | Neg | 0/2 |  | Neg | 0/2 |  | Neg |
|  |  |  |  |  | Saliva | 38.8 | Neg | Neg |  |  | Neg |  |  |  |
| 26 (H130009) | M | 43 | France (French Guiana |  | Skin biopsy | > 40 | Neg | Neg | 0/4 |  | Neg | 0/4 |  | Neg |
|  |  |  |  |  | Saliva | > 40 | Neg | Neg |  |  | Neg |  |  |  |
|  |  |  |  |  | Saliva | 39.3 | Neg | Neg |  |  | Neg |  |  |  |
|  |  |  |  |  | Saliva | 38.8 | Neg | Neg |  |  | Neg |  |  |  |
| 27 (H140001) | M | 48 | France (French Guiana |  | Skin biopsy | > 40 | Neg | Neg | 0/4 |  | Neg | 0/4 |  | Neg |
|  |  |  |  |  | Saliva | > 40 | Neg | Neg |  |  | Neg |  |  |  |
|  |  |  |  |  | Saliva | > 40 | Neg | Neg |  |  | Neg |  |  |  |
|  |  |  |  |  | Saliva | > 40 | Neg | Neg |  |  | Neg |  |  |  |
| 28 (H140004) | M | 57 | France (ex Mali) |  | Skin biopsy | > 40 | Neg | Pos (77.2) | 2/14 |  | Pos | 11/14 |  | Pos |
|  |  |  |  |  | CSF | 40 | Neg | Neg |  |  | Neg |  |  |  |
|  |  |  |  |  | Saliva | > 40 | Neg | Neg |  |  | Pos |  |  |  |
|  |  |  |  |  | Saliva | > 40 | Neg | Neg |  |  | Pos |  |  |  |
|  |  |  |  |  | Saliva | > 40 | Neg | Neg |  |  | Pos |  |  |  |
|  |  |  |  |  | Saliva | > 40 | Neg | Neg |  |  | Pos |  |  |  |
|  |  |  |  |  | Saliva | > 40 | Neg | Neg |  |  | Pos |  |  |  |
|  |  |  |  |  | Saliva | > 40 | Neg | Neg |  |  | Pos |  |  |  |
|  |  |  |  |  | Saliva | > 40 | Neg | Neg |  |  | Pos |  |  |  |
|  |  |  |  |  | Saliva | > 40 | Neg | Neg |  |  | Pos |  |  |  |
|  |  |  |  |  | Saliva | > 40 | Neg | Neg |  |  | Neg |  |  |  |
|  |  |  |  |  | Saliva | > 40 | Neg | Neg |  |  | Neg |  |  |  |
|  |  |  |  |  | Skin biopsy | > 40 | Neg | Pos (77.2) |  |  | Pos |  |  |  |
|  |  |  |  |  | Bronchial secretions aspirate | > 40 | Neg | Neg |  |  | Pos |  |  |  |
| 29 (H140005) | M | 48 | France |  | Skin biopsy | > 40 | Neg | Neg | 0/8 |  | Neg | 0/8 |  | Neg |
|  |  |  |  |  | Saliva | 38.9 | Neg | Neg |  |  | Neg |  |  |  |
|  |  |  |  |  | Saliva | > 40 | Neg | Neg |  |  | Neg |  |  |  |
|  |  |  |  |  | Saliva | > 40 | Neg | Neg |  |  | Neg |  |  |  |
|  |  |  |  |  | Skin biopsy | > 40 | Neg | Neg |  |  | Neg |  |  |  |
|  |  |  |  |  | Saliva | > 40 | Neg | Neg |  |  | Neg |  |  |  |
|  |  |  |  |  | Saliva | > 40 | Neg | Neg |  |  | Neg |  |  |  |
|  |  |  |  |  | Saliva | > 40 | Neg | Neg |  |  | Neg |  |  |  |
| 30 (H140006) | M | 19 | France (French Guiana |  | Skin biopsy | > 40 | Neg | Neg | 0/4 |  | Neg | 0/4 |  | Neg |
|  |  |  |  |  | Saliva | > 40 | Neg | Neg |  |  | Neg |  |  |  |
|  |  |  |  |  | Saliva | > 40 | Neg | Neg |  |  | Neg |  |  |  |
|  |  |  |  |  | Saliva | > 40 | Neg | Neg |  |  | Neg |  |  |  |
| 31 (H140007) | F | 55 | Italia (from Kenya) |  | Skin biopsy | 33.9 | Pos | Neg | 3/3 |  | Neg | 2/3 |  | Pos |
|  |  |  |  |  | CSF | 25.2 | Pos | Neg |  |  | Pos |  |  |  |
|  |  |  |  |  | CSF | 25.4 | Pos | Neg |  |  | Pos |  |  |  |
| 32 (04030PHI) |  |  | Philippines |  | Brain biopsy | 16.1 | Pos | ND | 1/1 |  | Pos | 1/1 |  | Pos |
|  |  |  |  |  |  |  |  |  |  |  |  |  |  |  |
| 33 (9702IND) | F | 30 | France (ex India) |  | Brain biopsy | 28.2 | Pos | ND | 1/1 |  | Pos | 1/1 |  | Pos |
|  |  |  |  |  |  |  |  |  |  |  |  |  |  |  |
| 34 (04030PHI) |  |  | Philippines |  | Brain biopsy | 16.1 | Pos | ND | 1/1 |  | Pos | 1/1 |  | Pos |
|  |  |  |  |  |  |  |  |  |  |  |  |  |  |  |
| 35 | ? | ? | Morocco |  | Brain biopsy | > 40 | Neg | Neg | 0/1 |  | Neg | 0/1 |  | Neg |
| 36 | 39 | M | Morocco |  | Brain biopsy | 22.3 | Pos | Neg | 1/1 |  | Pos | 1/1 |  | Pos |
| 37 | 6 | F | Morocco |  | Brain biopsy | 18.9 | Pos | Neg | 1/1 |  | Pos | 1/1 |  | Pos |
| 38 | ? | M | Morocco |  | Brain biopsy | 24.4 | Pos | Neg | 1/1 |  | Pos | 1/1 |  | Pos |
| 39 | 12 | M | Morocco |  | Skin biopsy | 36 | Pos | ND | 1/1 |  | Pos | 1/1 |  | Pos |
| 40 | 29 | F | Morocco |  | Skin biopsy | 35.1 | Pos | ND | 1/1 |  | Pos | 1/1 |  | Pos |
| 41 | 58 | M | Morocco |  | Skin biopsy | 35.6 | Pos | ND | 1/1 |  | Pos | 1/1 |  | Pos |
| 42 | 47 | M | Morocco |  | Skin biopsy | 34 | Pos | ND | 1/1 |  | Pos | 1/1 |  | Pos |
| 43 | 59 | M | Morocco |  | Skin biopsy | 33.7 | Pos | ND | 1/1 |  | Pos | 1/1 |  | Pos |
| 44 (ACIP 029) | F | 46 | Cambodia |  | Brain biopsy | 19.6 | Pos | ND | 1/1 |  | Pos | 1/1 |  | Pos |
|  |  |  |  |  |  |  |  |  |  |  |  |  |  |  |
| 45 (ACIP 031) | F | 11 | Cambodia |  | Brain biopsy | 32.8 | Pos | ND | 1/1 |  | Pos | 1/1 |  | Pos |
|  |  |  |  |  |  |  |  |  |  |  |  |  |  |  |
| 46 (ACIP 034) | M | 50 | Cambodia |  | Brain biopsy | 18.1 | Pos | ND | 1/1 |  | Pos | 1/1 |  | Pos |
|  |  |  |  |  |  |  |  |  |  |  |  |  |  |  |
| 47 (ACIP 36) | M | 33 | Cambodia |  | Brain biopsy | 17.5 | Pos | ND | 1/1 |  | Pos | 1/1 |  | Pos |
|  |  |  |  |  |  |  |  |  |  |  |  |  |  |  |
| 48 (ACIP 02) | F | 11 | Cambodia |  | Skin biopsy | > 40 | Neg | ND | 2/3 |  | Pos (weak) | 3/3 |  | Pos |
|  |  |  |  |  | Skin biopsy | 36.1 | Pos | ND |  |  | Pos |  |  |  |
|  |  |  |  |  | Skin biopsy | 35.9 | Pos | ND |  |  | Pos |  |  |  |
| 49 (ACIP 03) | M | 30 | Cambodia |  | Skin biopsy | 34.3 | Pos | ND | 2/2 |  | Pos | 2/2 |  | Pos |
|  |  |  |  |  | Skin biopsy | 31.2 | Pos | ND |  |  | Pos |  |  |  |
| 50 (ACIP 04) | M | 32 | Cambodia |  | Skin biopsy | 34.5 | Pos | ND | 1/1 |  | Pos | 1/1 |  | Pos |
|  |  |  |  |  |  |  |  |  |  |  |  |  |  |  |
| 51 (ACIP 05) | F | 47 | Cambodia |  | Skin biopsy | 28.7 | Pos | ND | 1/1 |  | Pos | 1/1 |  | Pos |
| 52 (ACIP 07) | F | 30 | Cambodia |  | Skin biopsy | 33.1 | Pos | ND | 1/1 |  | Pos | 1/1 |  | Pos |
| 53 (ACIP 08) | F | 8 | Cambodia |  | Skin biopsy | 32.2 | Pos | ND | 1/1 |  | Pos | 1/1 |  | Pos |
| 54 (ACIP 10) | M | 8 | Cambodia |  | Skin biopsy | 31.2 | Pos | ND | 2/2 |  | Pos | 2/2 |  | Pos |
|  |  |  |  |  | Skin biopsy | 30.2 | Pos | ND |  |  | Pos |  |  |  |
| 55 (ACIP 11) | M | 27 | Cambodia |  | Skin biopsy | 30.8 | Pos | ND | 2/2 |  | Pos | 2/2 |  | Pos |
|  |  |  |  |  | Skin biopsy | 30.8 | Pos | ND |  |  | Pos |  |  |  |
| 56 (ACIP 12) | M | 27 | Cambodia |  | Skin biopsy | > 40 | Neg | ND | 1/2 |  | Neg | 1/2 |  | Pos |
|  |  |  |  |  | Skin biopsy | 34.2 | Pos | ND |  |  | Pos (weak) |  |  |  |
| 57 (ACIP 13) | M | 13 | Cambodia |  | Skin biopsy | 33.8 | Pos | ND | 2/2 |  | Pos (weak) | 2/2 |  | Pos |
|  |  |  |  |  | Skin biopsy | 34.5 | Pos | ND |  |  | Pos |  |  |  |
| 58 (ACIP 15) | M | 20 | Cambodia |  | Skin biopsy | 32.2 | Pos | ND | 1/1 |  | Pos | 1/1 |  | Pos |
| 59 (ACIP 18) | M | 5 | Cambodia |  | Skin biopsy | 32.7 | Pos | ND | 2/2 |  | Pos | 2/2 |  | Pos |
|  |  |  |  |  | Skin biopsy | 31.7 | Pos | ND |  |  | Pos |  |  |  |
| 60 (ACIP 19) | F | 60 | Cambodia |  | Skin biopsy | 14.1 | Pos | ND | 2/2 |  | Pos | 2/2 |  | Pos |
|  |  |  |  |  | Skin biopsy | 30 | Pos | ND |  |  | Pos |  |  |  |
| 61 (ACIP 22) | M | 39 | Cambodia |  | Skin biopsy | 30.8 | Pos | ND | 2/2 |  | Pos | 2/2 |  | Pos |
|  |  |  |  |  | Skin biopsy | 32.9 | Pos | ND |  |  | Pos |  |  |  |
| 62 (ACIP 23) | F | 14 | Cambodia |  | Skin biopsy | 32.1 | Pos | ND | 2/2 |  | Pos | 2/2 |  | Pos |
|  |  |  |  |  | Skin biopsy | 31.8 | Pos | ND |  |  | Pos |  |  |  |
| 63 (ACIP 24) | F | 32 | Cambodia |  | Skin biopsy | 32.8 | Pos | ND | 2/2 |  | Pos | 2/2 |  | Pos |
|  |  |  |  |  | Skin biopsy | 32.7 | Pos | ND |  |  | Pos |  |  |  |
| 64 (ACIP 25) | M | 13 | Cambodia |  | Skin biopsy | 32.6 | Pos | ND | 2/2 |  | Pos | 2/2 |  | Pos |
|  |  |  |  |  | Skin biopsy | 32 | Pos | ND |  |  | Pos |  |  |  |
| 65 (ACIP 28) | M | 8 | Cambodia |  | Skin biopsy | 29.5 | Pos | ND | 2/2 |  | Pos | 2/2 |  | Pos |
|  |  |  |  |  | Skin biopsy | 29.6 | Pos | ND |  |  | Pos |  |  |  |

^a^ These results are a compilation obtained from three independant centres : National Reference Centre for Rabies (NRC-R, Institut Pasteur, Paris, France) for patients 1 to 34, Institut Pasteur of Morocco (IPM) for patients 35 to 43, Institut Pasteur of Cambodia (IPC) for patients 44 to 65.

^b^ M : male, F : female.

^c^ Mean value of two technical replicates.

^d^ Pos : positive, Neg : negative

^e^ ND : not done

^f^ Tm : hybridization temperature
