# Supplementary material for: Dual Combined Real-Time Reverse Transcription Polymerase Chain Reaction Assay for the Diagnosis of Lyssavirus Infection
Source: PLoS Negl Trop Dis. 2016 Jul 5;10(7):e0004812. doi: 10.1371/journal.pntd.0004812 (PMC4933377; doi:10.1371/journal.pntd.0004812)
Supplement: S5 Table — (DOCX) [file pntd.0004812.s006.docx]

**S5 Table : Description of samples used for the *post-mortem* diagnosis of animal rabies and results of the analytical sensitivity of the combo RT-qPCR assay.**

| **Species** | **Isolate^a^** | **Sample^b^** | **Host^c^** | **Origin^d^** | **Year of isolation^d^** | **Pan-RABV (TaqMan)** | | **Pan-lyssa (SYBR Green)** | |
| --- | --- | --- | --- | --- | --- | --- | --- | --- | --- |
|  |  |  |  |  |  | **Result^e^** | **Mean value (Cq)^efg^** | **Result^e^** | **Mean value (Tm)^efh^** |
| ABLV | 9810AUS | M | Bat (?) | Australia | - | ND | ND | Pos | 78.3 |
| BBLV | 13001FRA | P | Bat (*Myotis nattereri*) | France | 2013 | Neg | N/A | Pos | 77.2 |
| DUVV | 9020SA | M | Bat (?) | South Africa | - | ND | ND | Pos | 77.3 |
| DUVV | 86132SA | M | Human | South Africa | 1971 | Neg | N/A | Pos | 77 |
| DUVV | 94286SA | M | Bat *(Miniopterus* sp.) | South Africa | 1981 | Neg | N/A | Pos | 77 |
| EBLV-1 | 122938* | M | Bat (*Eptesicus serotinus*) | France | 2002 | Pos | 30.6 | Pos | 78.3 |
| EBLV-1 | 02007DEN | M | Bat (?) | Denmark | 1993 | ND | ND | Pos | 78.3 |
| EBLV-1 | 02031FRA | M | Bat (*Eptesicus serotinus*) | France | 2002 | ND | ND | Pos | 79.1 |
| EBLV-1 | 03011FRA | P | Cat | France | 2003 | ND | ND | Pos | 74.8 |
| EBLV-1 | 06001FRA | P | Bat (*Eptesicus serotinus*) | France | 2006 | ND | ND | Pos | 78.6 |
| EBLV-1 | 06002FRA | P | Bat (*Eptesicus serotinus*) | France | 2006 | ND | ND | Pos | 78.46 |
| EBLV-1 | 07240FRA | P | Cat | France | 2007 | ND | ND | Pos | 79 |
| EBLV-1 | 8919FRA | M | Bat (*Eptesicus serotinus*) | France | 1989 | ND | ND | Pos | 78.9 |
| EBLV-1 | 9397GER | M | Bat (?) | Germany | - | ND | ND | Pos | 78.4 |
| EBLV-1 | 9399GER | M | Bat (*Eptesicus serotinus*) | Germany | 1982 | ND | ND | Pos | 78.7 |
| EBLV-1 | 9439GER | M | Bat (*Eptesicus serotinus*) | Germany | 1989 | ND | ND | Pos | 78.6 |
| EBLV-1 | 9366HOL | M | Bat (*Eptesicus serotinus*) | The Netherlands | 1992 | ND | ND | Pos | 78.7 |
| EBLV-1 | 9376HOL | M | Bat (*Eptesicus serotinus*) | The Netherlands | 1993 | ND | ND | Pos | 78.6 |
| EBLV-1 | 94115HOL | M | Bat (*Eptesicus serotinus*) | The Netherlands | 1989 | ND | ND | Pos | 78.8 |
| EBLV-1 | 9480HOL | M | Bat (*Eptesicus serotinus*) | The Netherlands | 1987 | ND | ND | Pos | 78.8 |
| EBLV-1 | 9443UKR | M | Bat (*Vespertilio murinus)* | Ukraine | 1987 | ND | ND | Pos | 78.6 |
| EBLV-1 | 03002FRA | M | Bat (*Eptesicus serotinus*) | France | 2003 | Neg | N/A | Pos | 79.2 |
| EBLV-1 | 04032FRA | P | Bat (*Eptesicus serotinus*) | France | 2004 | Neg | N/A | Pos | 78.6 |
| EBLV-2 | RV178* | M | Bat (*Myotis daubentonii*) | United Kingdom | 2004 | Pos | 31.9 | Pos | 74.7 |
| EBLV-2 | 9007FIN | M | Human | Finland | 1985 | ND | ND | Pos | 77.6 |
| EBLV-2 | 02055SWI | M | Bat (*Myotis daubentonii*) | Switzerland | 1992 | ND | ND | Pos | 74.6 |
| EBLV-2 | 9337SWI | M | Bat (*Myotis daubentonii*) | Switzerland | 1993 | ND | ND | Pos | 75.3 |
| EBLV-2 | 9018HOL | M | Bat (*Myotis dasycneme*) | The Netherlands | 1986 | ND | ND | Pos | 75.8 |
| EBLV-2 | 02053SWI | M | Bat (*Myotis daubentonii*) | Switzerland | 2002 | Neg | N/A | Pos | 75.3 |
| LBV | 8620RCA | M | Bat (*Micropteropus pusillus*) | Central African Republic | 1974 | Neg | N/A | Pos | 78.5 |
| LBV | 8619NIG | M | Bat (*Eidolon helvum*) | Nigeria | 1956 | Neg | N/A | Pos | 78.6 |
| MOKV | 86100CAM | M | Shrew | Cameroon | 1974 | ND | ND | Pos | 78.7 |
| MOKV | 86101RCA | M | Rodent | Central African Republic | 1981 | Neg | N/A | Pos | 79.5 |
| MOKV | 8720SA | M | Cat | South Africa | - | Neg | N/A | Pos | 79 |
| RABV | 96178POL | P | Red fox | Poland | 1994 | Pos | 12.25 | ND | ND |
| RABV | 9507CZE | M | Vaccine strain | Czech Republic | 1994 | Pos | 13.9 | ND | ND |
| RABV | O0221516** | P | Dog | Cambodia | - | Pos | 13.91 | ND | ND |
| RABV | 9915BIR | M | Dog | Myanmar | 1999 | Pos | 14.26 | ND | ND |
| RABV | 02047CHI | P | Dog | China | 1994 | Pos | 14.56 | ND | ND |
| RABV | 9910LAO | M | Dog | Lao | 1999 | Pos | 14.56 | ND | ND |
| RABV | P0806514** | P | Dog | Cambodia | - | Pos | 14.59 | ND | ND |
| RABV | 9142EST | M | Raccoon dog | Estonia | 1991 | Pos | 14.75 | ND | ND |
| RABV | O0625524** | P | Dog | Cambodia | - | Pos | 15.06 | ND | ND |
| RABV | O0831550** | P | Dog | Cambodia | - | Pos | 15.25 | ND | ND |
| RABV | 9616FRA | P | Sheep | France | 1996 | Pos | 15.32 | ND | ND |
| RABV | O0217545** | P | Dog | Cambodia | - | Pos | 15.39 | ND | ND |
| RABV | P0623534** | P | Dog | Cambodia | - | Pos | 15.42 | ND | ND |
| RABV | O0716535** | P | Dog | Cambodia | - | Pos | 15.8 | ND | ND |
| RABV | O0518537** | P | Dog | Cambodia | - | Pos | 15.83 | ND | ND |
| RABV | T1221671** | P | Dog | Cambodia | - | Pos | 15.91 | ND | ND |
| RABV | 9722POL | P | Raccoon dog | Poland | 1996 | Pos | 15.91 | ND | ND |
| RABV | O1025556** | P | Dog | Cambodia | - | Pos | 16.01 | ND | ND |
| RABV | P0416516** | P | Dog | Cambodia | - | Pos | 16.17 | ND | ND |
| RABV | 11028CAM | P | Dog | Cameroon | 2009 | Pos | 16.25 | ND | ND |
| RABV | 11040MAR | P | Dog | Morocco | 2011 | Pos | 16.25 | ND | ND |
| RABV | 02056GAB | P | Dog | Gabon | 2002 | Pos | 16.28 | ND | ND |
| RABV | P0407547** | P | Dog | Cambodia | - | Pos | 16.29 | ND | ND |
| RABV | O1119516** | P | Dog | Cambodia | - | Pos | 16.48 | ND | ND |
| RABV | 11020CAM | P | Dog | Cameroon | 2009 | Pos | 16.5 | ND | ND |
| RABV | P1114533** | P | Dog | Cambodia | - | Pos | 16.71 | ND | ND |
| RABV | 01017VNM | M | Dog | Vietnam | 2011 | Pos | 16.79 | ND | ND |
| RABV | 08342MAR | P | Dog | Morocco | 2008 | Pos | 16.86 | ND | ND |
| RABV | P1110521** | P | Dog | Cambodia | - | Pos | 16.87 | ND | ND |
| RABV | 04035AFG | P | Dog | Afghanistan | 2004 | Pos | 16.89 | ND | ND |
| RABV | P0428506** | P | Dog | Cambodia | - | Pos | 16.89 | ND | ND |
| RABV | 9914CBG | M | Dog | Cambodia | 1997 | Pos | 16.93 | ND | ND |
| RABV | P0822526** | P | Dog | Cambodia | - | Pos | 16.95 | ND | ND |
| RABV | 8623GAB | M | Dog | Gabon | - | Pos | 16.95 | ND | ND |
| RABV | 8690CHI | M | Suede | China | 1985 | Pos | 16.99 | ND | ND |
| RABV | P0709509** | P | Dog | Cambodia | - | Pos | 17.18 | ND | ND |
| RABV | 8908GUY | P | Bovine | French Guiana | 1989 | Pos | 17.2 | ND | ND |
| RABV | 94289RWA | P | Dog | Rwanda | 1994 | Pos | 17.64 | ND | ND |
| RABV | 11009NEP | P | Dog | Nepal | 2010 | Pos | 17.69 | ND | ND |
| RABV | 9737POL | P | Raccoon dog | Poland | 1997 | Pos | 17.83 | ND | ND |
| RABV | 11018CAM | P | Dog | Cameroon | 2010 | Pos | 17.94 | ND | ND |
| RABV | 9104USA | M | Skunk | USA | 1991 | Pos | 18.02 | ND | ND |
| RABV | 9212ALL | M | Red fox | Germany | 1991 | Pos | 18.15 | ND | ND |
| RABV | 8687FRA | P | Dog | France | 1983 | Pos | 18.78 | ND | ND |
| RABV | 8801CAM | P | Dog | Cameroon | 1987 | Pos | 18.95 | ND | ND |
| RABV | 02052AFG | P | Dog | Afghanistan | 2002 | Pos | 19.56 | ND | ND |
| RABV | 9021TCH | P | Dog | Chad | 1990 | Pos | 19.76 | ND | ND |
| RABV | 9140FRA | M | Bovine | French Guiana | 1991 | Pos | 19.98 | ND | ND |
| RABV | 8689MAU | P | Camel | Mauritania | 1986 | Pos | 20.3 | ND | ND |
| RABV | 8670NIG | M | Unknown | Nigeria | - | Pos | 20.34 | ND | ND |
| RABV | 8697BEN | P | Cat | Benin | 1986 | Pos | 20.74 | ND | ND |
| RABV | O0405536** | P | Dog | Cambodia | - | Pos | 21.09 | ND | ND |
| RABV | 9704ARG | M | Bat (*Tadarida brasiliensis*) | Argentina | 1997 | Pos | 21.39 | ND | ND |
| RABV | O1227521** | P | Dog | Cambodia | - | Pos | 21.55 | ND | ND |
| RABV | 8668CI | M | ? | Ivory Coast | - | Pos | 21.69 | ND | ND |
| RABV | 9912CBG | M | Dog | Cambodia | 1998 | Pos | 21.95 | ND | ND |
| RABV | 9701FRA | P | Bovine | French Guiana | 1997 | Pos | 22.25 | ND | ND |
| RABV | 96321IRA | P | Jackal | Iran | 1996 | Pos | 23.01 | ND | ND |
| RABV | 86107YOU | M | Red fox | Yougoslavia (formerly) | 1976 | Pos | 23.33 | ND | ND |
| RABV | 02034MAR | P | Dog | Morocco | 2002 | Pos | 23.65 | ND | ND |
| RABV | 08338GAM | P | Dog | Gambia | 2008 | Pos | 24.56 | ND | ND |
| RABV | 9135OMA | M | Fox | Oman | 1990 | Pos | 26.42 | ND | ND |
| RABV | 04034ARS | P | Red fox | Saudi Arabia | 2004 | Pos | 20.58 | ND | ND |
| RABV | 11008NEP | P | Goat | Nepal | 2010 | Pos | 27.74 | ND | ND |
| RABV | 9013NIG | P | Dog | Niger | 1990 | Pos | 29.01 | ND | ND |
| RABV | 9901NEP | M | Dog | Nepal | 1998 | Pos | 29.17 | ND | ND |
| RABV | 01003MAR | P | Dog | Morocco | 2001 | Pos | 29.55 | ND | ND |
| RABV | 10007RCA | P | Dog | Central African  Republic | 2010 | Pos | 31.35 | ND | ND |
| RABV | 96330FRA | P | Bovine | French Guiana | 1996 | Pos | 32.27 | ND | ND |
| RABV | 09032AFG | P | Dog | Afghanistan | 2009 | Pos | 33.09 | ND | ND |
| RABV | 9141RUS | M | Arctic fox | Russia | 1988-90 | Pos | 34.85 | ND | ND |
| RABV | 94288FRA | P | Red fox | France | 1994 | Pos | 9.88 | ND | ND |
| RABV | 87012MAR | P | Dog | Morocco | 1986 | Pos | 13.48 | Neg | 77.4 |
| RABV | 08225ALG | P | Dog | Algeria | 2002 | Pos | 15.02 | Neg | N/A |
| RABV | 9024GUI | P | Dog | Guinea | 1990 | Pos | 18.21 | Neg | 78.8 |
| RABV | 9233GAB | M | Dog | Gabon | 1992 | Pos | 19.61 | Neg | N/A |
| RABV | 03001FRA | P | Dog | French Guiana | 2003 | Pos | 20.56 | Neg | N/A |
| RABV | 9811CHI | P | Dog | China | 1998 | Pos | 21.72 | Neg | N/A |
| RABV | 14011SEN | P | Cat | Senegal | 2004 | Neg | N/A | Neg | N/A |
| RABV | 07059CI | P | Dog | Ivory Coast | 2007 | Pos | 25 | Pos | 78.1 |
| RABV | CVS27 14-10* | M | Laboratory strain | - | - | Pos | 11.8 | Pos | 76.7 |
| RABV | GS7* | M | Red fox | France | 1986 | Pos | 12.5 | Pos | 76.8 |
| RABV | 8917MAR | M | Dog | Morocco | 1989 | Pos | 12.56 | Pos | 77.6 |
| RABV | 13002FRA | P | Cat | France (ex Morocco) | 2013 | Pos | 12.8 | Pos | 76.9 |
| RABV | Cn Viv Estonie 10-12* | M | Raccoon dog | Estonia | 2006 | Pos | 13.5 | Pos | 77.6 |
| RABV | Macedonia 37-12 * | M | Red fox | Republic of Macedonia | 2011 | Pos | 14.1 | Pos | 76.8 |
| RABV | 9531ETH | P | Bovine | Ethiopia | 1987 | Pos | 14.65 | Pos | 76.8 |
| RABV | 07141RCA | P | Dog | Central African Republic | 2003 | Pos | 15.01 | Pos | 77.7 |
| RABV | 03009MAR | M | Dog | Morocco | 2003 | Pos | 15.05 | Pos | 78.1 |
| RABV | 8684GRO | P | Arctic fox | Greenland | 1981 | Pos | 16.45 | Pos | 78.4 |
| RABV | 9131MAU | P | Dog | Mauritania | 1991 | Pos | 16.5 | Pos | 78.1 |
| RABV | 9103USA | M | Bat (?) | USA | 1991 | Pos | 17.15 | Pos | 77.9 |
| RABV | 8913CI | M | Dog | Ivory Coast | 1989 | Pos | 17.64 | Pos | 78.2 |
| RABV | 9010NIG | P | Dog | Niger | 1990 | Pos | 18.15 | Pos | 77.1 |
| RABV | 02002LAO | M | Dog | Lao | 2002 | Pos | 18.22 | Pos | 77.1 |
| RABV | GS7 diluted 1/30* | M | Red fox | France | 1986 | Pos | 18.3 | Pos | 76.8 |
| RABV | 8676TUN | M | Human | Tunisia | 1986 | Pos | 18.43 | Pos | 77.3 |
| RABV | GS7 diluted 1/50* | M | Red fox | France | 1986 | Pos | 18.7 | Pos | 76.8 |
| RABV | 9027BUR | P | Jackal | Burundi | 1990 | Pos | 19.08 | Pos | 78.1 |
| RABV | 9311MAU | M | Dog | Mauritania | 1993 | Pos | 20.57 | Pos | 77.7 |
| RABV | 8912CI | M | Dog | Ivory Coast | 1989 | Pos | 20.89 | Pos | 78.01 |
| RABV | 9011NIG | P | Dog | Niger | 1990 | Pos | 21.18 | Pos | 78.1 |
| RABV | 8718NIG | M | Dog | Niger | 1975 (1987) | Pos | 21.37 | Pos | 77.8 |
| RABV | 9105USA | M | Fox | USA | 1990 | Pos | 21.44 | Pos | 78.7 |
| RABV | 8717IRA | P | Jackal | Iran | 1984 | Pos | 21.55 | Pos | 77 |
| RABV | 9009NIG | P | Dog | Niger | 1990 | Pos | 21.72 | Pos | 77.6 |
| RABV | 02008CHI | P | Dog | China | - | Pos | 21.93 | Pos | 78.2 |
| RABV | 8642ETH | P | Dog | Ethiopia | - | Pos | 22.13 | Pos | 76.7 |
| RABV | 09037RCA | P | Dog | Central African Republic | 2009 | Pos | 22.14 | Pos | 78.4 |
| RABV | 03003INDO | M | Dog | Indonesia | 2003 | Pos | 23.26 | Pos | 79.2 |
| RABV | 8911CI | M | Dog | Ivory Coast | 1989 | Pos | 24.47 | Pos | 78.1 |
| RABV | 9218TCH | M | Dog | Chad | 1992 | Pos | 24.61 | Pos | 77.1 |
| RABV | 9026CI | M | Dog | Ivory Coast | 1990 | Pos | 25.61 | Pos | 77.9 |
| RABV | 04033MAD | M | Human | Madagascar | 2004 | Pos | 27.44 | Pos | 77.7 |
| RABV | 9238CI | P | Dog | Ivory Coast | 1992 | Pos | 27.83 | Pos | 77.9 |
| RABV | 8732TCH | P | Dog | Chad | 1987 | Pos | 32.49 | Pos | 78.6 |
| RABV | 14013SEN | P | Dog | Senegal | 2011 | Neg | N/A | Pos | 77.7 |
| RABV | 14014SEN | P | Dog | Senegal | 2011 | Neg | N/A | Pos | 77.7 |
| RABV | 14016BOS | P | Wild cat | Botswana | 2009 | Pos | 31.33 | Pos | 77.6 |
| RABV | 14017BOS | P | Honey badger | Botswana | 2009 | Pos | 36.37 | Neg | 77.5 |
| RABV | 14018SA | P | Cat | South Africa | 2000 | Pos | 20.29 | Pos | 77.3 |

^a^ : * : Samples tested at the NRC-R, Institut Pasteur, Paris, during its participation in the framework of the international interlaboratory trial organized by the European Union reference laboratory for rabies, ** : Samples tested at the Institut Pasteur du Cambodge, Cambodia. All other samples were tested at the NRC-R, Institut Pasteur, Paris

^b^ M : suckling newborn mouse brain sample, P : primary brain sample

^c^ ?: Species not available

^d^ - : No information available

^e^ Pos : positive, Neg : negative, ND : not done

^f^ N/A : no C_q_ or Tm value available

^g^ Mean Cq value from duplicates

^h^ Mean Tm value from duplicates
