# Supplementary material for: Dual Combined Real-Time Reverse Transcription Polymerase Chain Reaction Assay for the Diagnosis of Lyssavirus Infection
Source: PLoS Negl Trop Dis. 2016 Jul 5;10(7):e0004812. doi: 10.1371/journal.pntd.0004812 (PMC4933377; doi:10.1371/journal.pntd.0004812)
Supplement: S6 Table — (DOCX) [file pntd.0004812.s007.docx]

|  |  | **Pan-RABV (pos no./total no.)** | | | **Sensitivity (%)** | **Specificity (%)** |
| --- | --- | --- | --- | --- | --- | --- |
|  |  | **Morocco^a^** | **Cambodia^a^** | **Total** |  |  |
| Sample | Saliva | ND^b^ | 0/5 | 0/5 | 100 | 100 |
|  | Brain biopsy | 3/4  (Cq =21.86 ± 2.85) | 4/4  (Cq =22.53 ± 7) | 7/8  (Cq =22.24 ± 5.37) | 100 | 100 |
|  | Skin biopsy | 5/5  (Cq =34.9 ± 1.13) | 30/35  (Cq =32.31 ± 3.08) | 35/40  (Cq =32.68 ± 3.02) | 96.8 | 100 |
|  | Total | 8/8  (Cq =30 ± 6.73) | 34/44  (Cq =31.16 ± 4.85) | 42/52  (Cq =30.94 ± 5.24) | 97.7 | 100 |
|  |  |  |  |  |  |  |

**S6  Table**: Results of the implementation of the pan-RABV RT-qPCR in local settings of two national reference laboratories for rabies localized in Morocco and in Cambodia.

^a^ The pan-RABV RT-qPCR assay was performed with an Applied Biosystems 7500 Real-Time PCR System (Life Technologies, Saint Aubin, France) in Morocco and with a iQ5 real-time PCR detection system (BioRad, Marnes-la-Coquette, France) in Cambodia.

^b^ ND : Not done
