# Supplementary material for: Dual Combined Real-Time Reverse Transcription Polymerase Chain Reaction Assay for the Diagnosis of Lyssavirus Infection
Source: PLoS Negl Trop Dis. 2016 Jul 5;10(7):e0004812. doi: 10.1371/journal.pntd.0004812 (PMC4933377; doi:10.1371/journal.pntd.0004812)
Supplement: S7 Table — (DOCX) [file pntd.0004812.s008.docx]

| **Sample** | **Lyssavirus species** | **Strain** | **FAT^a^** | **RT-hnPCR^a^** |  | **Dual combined RT-qPCR results^a^**  **(Pos no. / Tested nb.)** | |
| --- | --- | --- | --- | --- | --- | --- | --- |
|  |  |  |  |  |  | **Pan-rabies (TaqMan)** | **Pan-lyssa result**  **(SYBR Green)** |
|  |  |  |  |  |  |  |  |
| 1 | RABV | CVS 27 14-10 | Pos | Pos |  | Pos (Cq=11.8) | Pos |
| 2 | RABV | Cn Viv Estonie 10-12 | Pos | Pos |  | Pos (Cq = 13.5) | Pos |
| 3 | RABV | Macedonia 37-12 | Pos | Pos |  | Pos (Cq = 14.1) | Pos |
| 4 | EBLV-1 | 122938 | Pos | Pos |  | Pos (Cq = 31.6) | Pos |
| 5 | EBLV-2 | EBL2 RV1787 | Pos | Pos |  | Pos (Cq = 31.9) | Pos |
| 6 | RABV | GS7 | Pos | Pos |  | Pos (Cq = 12.5) | Pos |
| 7 | RABV | GS7  (1/30) | Pos | Pos |  | Pos (Cq = 18.3) | Pos |
| 8 | RABV | GS7 (1/50) | Pos | Pos |  | Pos (Cq = 18.7) | Pos |
| 9 | Negative | - | Neg | Neg |  | Neg | Neg |
|  |  |  |  |  |  |  |  |

**S7 Table: Evaluation of the combo RT-qPCR assay in an international interlaboratory trial**

^a^ Pos : positive, Neg : negative
