# Supplementary material for: Dual Combined Real-Time Reverse Transcription Polymerase Chain Reaction Assay for the Diagnosis of Lyssavirus Infection
Source: PLoS Negl Trop Dis. 2016 Jul 5;10(7):e0004812. doi: 10.1371/journal.pntd.0004812 (PMC4933377; doi:10.1371/journal.pntd.0004812)
Supplement: S1 Fig — (PDF) [file pntd.0004812.s009.pdf]

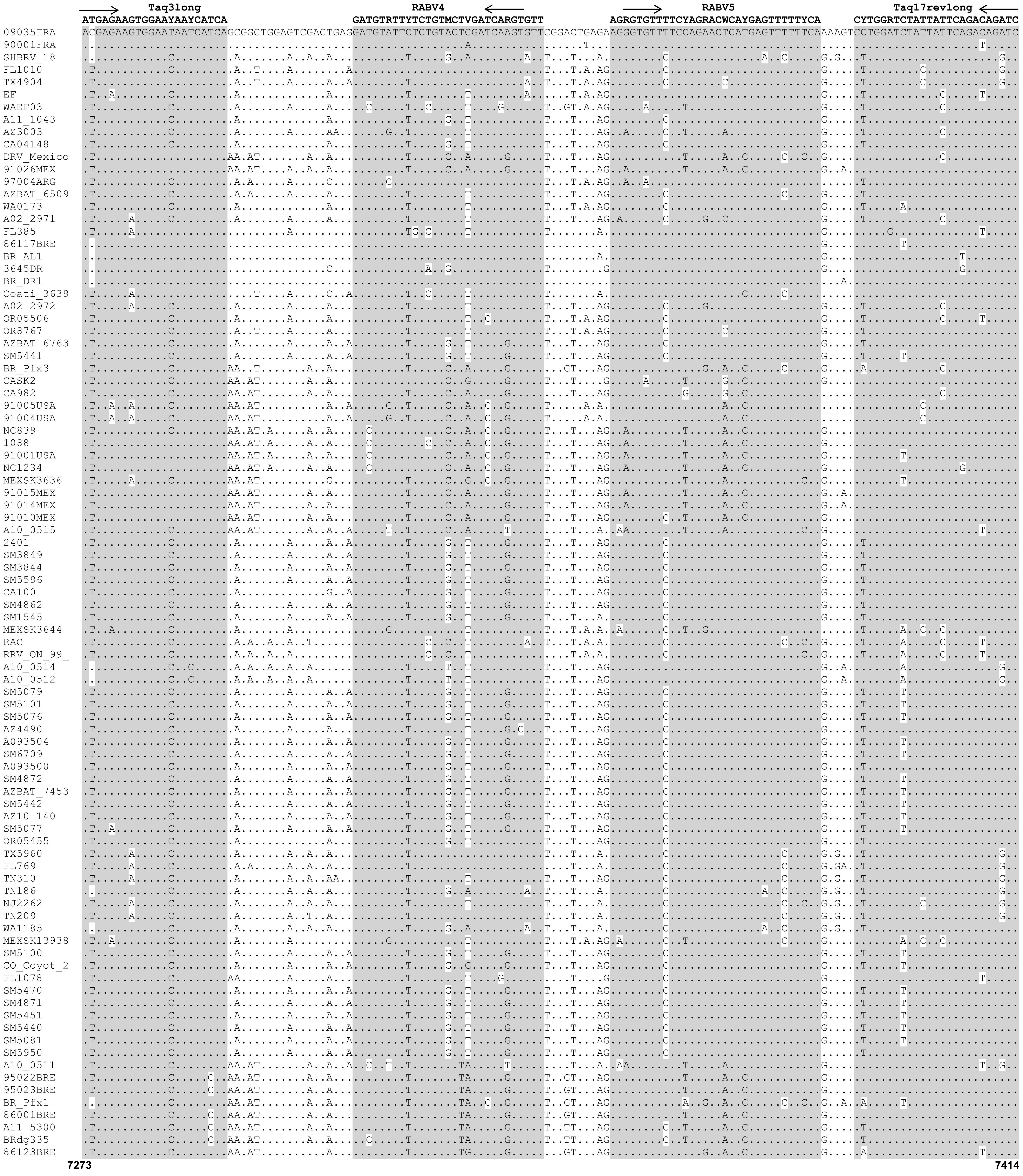

S1 Figure: Multiple alignment of the dataset of 91 partial polymerase sequences of New World RABV isolates, with nucleotide sequences and positions for primers Taq3long and Taq17revlong and for TaqMan hybridization probes RABV4 and RABV5 (pan-RABV RT-qPCR).

The oligonucleotide sequence of each primer and probe is indicated in bold, associated with its name and an arrow representing its sense. Identity to primer and probe sequences is highlighted in grey, respectively. Dots represent identity to the reference sequence 09035FRA. Position are indicated according to the reference sequence PV (GenBank accession number M13215). Asterisks indicated partial polymerase sequence obtained in this study. Description of the RABV isolates included in this multiple alignment can be retrieved in S2 Table.
